# The role of genetic polymorphisms in endolysosomal ion channels TPC2 and P2RX4 in cancer pathogenesis, prognosis, and diagnosis: a genetic association in the UK Biobank

**DOI:** 10.1038/s41525-021-00221-9

**Published:** 2021-07-12

**Authors:** Abeer F. Alharbi, John Parrington

**Affiliations:** 1 Department of Pharmacology, University of Oxford, Oxford, UK; 2 Pharmaceutical Sciences Department, College of Pharmacy, King Saud Bin Abdul-Aziz University for Health Sciences, Riyadh, Saudi Arabia

**Keywords:** Biomarkers, Cancer, Cancer genomics

## Abstract

Recent studies have implicated important roles for endolysosomal ion channels in cancer biology. We used UK Biobank data to characterise the relationships between genetic variants in two genes coding for endolysosomal ion channels—i.e. *TPCN2* and *P2RX4*—and cancer in terms of the definition of tumour types, susceptibility, and prognosis. We investigated these relationships at both global and local levels with regard to specific types of cancer, including malignant neoplasms of the brain, breast, bronchus, lung, colon, lymphoid and haematopoietic systems, skin, ovary, prostate, rectum, thyroid gland, lip, oral cavity, pharynx, and urinary tract. Apart from rs3829241 (*p* value < 0.05), all the genetic variants were in Hardy–Weinberg equilibrium. We included 468,436 subjects in the analysis and stratified them into two major cohorts: cancer-free controls (385,253) and cancer cases (83,183). For the first time, we report novel associations between genetic variants of *TPCN2* and *P2RX4* and cancer/cancer subtypes in the UK Biobank’s population. Genotype GG in *TPCN2* rs3750965 was significantly associated with a decreased risk of cancer and an increased risk of lip, oral cavity, and pharynx cancer and cancer recurrence in patients with prostate cancer, and genotypes GA/GG were associated with a significantly lower risk of developing various malignant neoplasms (involving melanoma, prostate, mesothelial, and soft tissues). rs35264875:TA was associated with a high risk of cancer at the global level, with subtypes of cancer at the local level (including breast, colon, prostate, and stated or presumed primary cancer of lymphoid, haematopoietic, and related tissue), and with a significantly low risk of cancer metastasis. rs72932540:GA was associated with a higher incidence of cancer/cancer subtypes (including breast, melanoma, and rectal cancer), and genotypes GA/GG were associated with an increased risk of prostate cancer. The *P2RX4* rs25644 allele GG was associated with a high risk of prostate cancer, whereas it was associated with a low risk of cancer recurrence in patients with prostate cancer. Genotypes GA/GG in rs28360472 were associated with an increased risk of breast, mesothelial, and soft tissue cancers but with a decreased risk of colon cancer. We also provide insights into the pathophysiological contributions made by these significant polymorphisms to cancer/cancer subtypes and their effects on expression or channel activity. Further investigations of these genetic variants could help identify novel cancer biomarkers and facilitate the development of new diagnostic and therapeutic strategies. This would constitute a further step towards personalised cancer care.

## Introduction

**Fig. 1:**
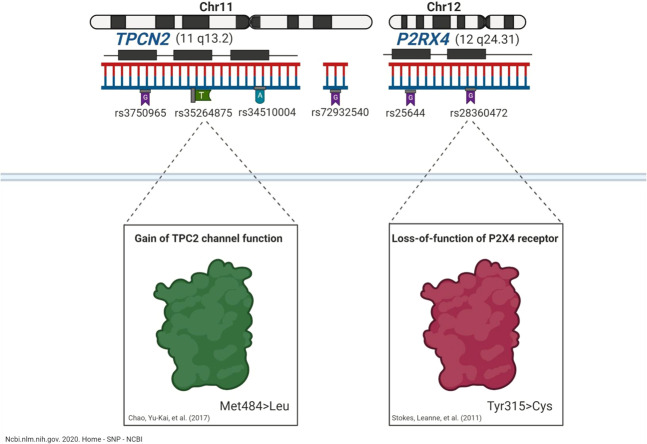
Schematic representation of genetic polymorphisms in endolysosomal ion channels TPC2 and P2RX4 that are included in our analysis. (The figure was created with BioRender.).

Cancer is a major public health problem and one of the leading causes of death worldwide. Cancer biomarkers have emerged as clinical tools that can enhance the efficiency of detection and guide the treatment of cancer patients by providing personalised therapy and information about expected cancer outcomes. The UK Biobank is a large-scale repository of clinical and genetic information from >500,000 individuals recruited from across the United Kingdom. Although a growing body of evidence underscores the biological significance of endolysosomal ion channels in cancer, from tumorigenesis to metastasis^1–3^, there is a paucity of human cancer data to decipher their potential translational and clinical values and their possible implications for cancer medicine genomics. This study focusses on two specific endolysosomal ion channels: the two-pore channel 2 (TPC2, also known as TPCN2) and the P2X4 ATP-activated cation channel (P2RX4)^4,5^. Recently, Böck et al. detailed some *TPCN2* genomic architecture, finding that *TPCN2* genetic variations are more common than other channels of the endolysosomal system (including TPC1, TRPML1, TRPML2, and TRPML3) on a global scale^6^. A recent RNA sequence analysis by Howarth et al. detected *TPCN2* exon 5 fused to *RSF1* exon 4 in the MDA-MB-175 cell line (in vitro model) of breast cancer^7^. A significant reduction in *TPCN2* expression was reported in metastatic compared to primary site patients with skin cutaneous melanoma using The Cancer Genome Atlas (TCGA) data^8^. rs72932540 is a variant located on ch 11:69,154,575, which is upstream at a distance of 105,643 from the transcription start site (TSS) of the *TPCN2* gene^9^ and has been reported to be associated with breast cancer^10^. Rapidly evolving significant biomedical evidence that links TPC2 to cancer^11^ and the highly reported genetic variations^6^ at the global level necessitate investigating the association between *TPCN2* polymorphisms and cancer in humans to provide insight into its potential applications as a biomarker in terms of the definition of tumour types, susceptibility, prognosis, and cancer outcomes. Emerging roles of P2X receptors in cancer biology have been implicated^12^, and pharmacological inhibition of P2RX4 has led to a reduction in cancer pain^13–16^. The recently identified biological significance of P2RX4 in prostate human biology by He et al.^17^ warrants further investigation in humans to discover the clinical genomic relevance of P2RX4 variation and prostate cancer at the phenotypic level, which is reflected in changes in expression or activity at the molecular level that have contributed to understanding P2RX4 roles in the pathophysiology of prostate cancer and their diagnostic and therapeutic applications. Our study characterises the relationship between genetic variants in *TPCN2* and *P2RX4* (shown in Fig. 1) and cancer both at a global level and in specific types of cancer that include malignant neoplasms of the brain, breast, bronchus, lung, colon, lymphoid and haematopoietic systems, skin, lip, oral cavity and pharynx, ovary, prostate, rectum, thyroid gland, and urinary tract in terms of cancer risk, disease recurrence, malignancy, and metastasis in the UK Biobank population. Here we discovered a novel association between polymorphisms in *TPCN2* and *P2RX4* in cancer at the global level for subtypes of cancer. We further investigated the potential impact of these significant genetic variants on channel expression/activity in similar biological/clinical contexts utilising publicly available bioinformatics tools.

## Results

### Participants’ characteristics

A total of 468,436 subjects were reviewed: 214,419 males (45.77%) and 254,017 (54.23%) females, the majority of whom were white. We identified 385,253 (82.24%) as cancer-free controls and 83,183 (17.76%) as cancer cases, and the median age at cancer diagnosis was 59.90 years (5.20–79.20) (see Table 1). This study included all types of tumours, but malignant neoplasm of the breast was the most common type (14.49%). In the following analysis, values were adjusted to take into account sex and ethnicity, as well as age where indicated.

**Table 1:** Subject characteristics and univariate analysis.

	UK Biobank ID	Total	Cancer cases	Cancer-free controls	*P* value
*Number (n, %)*		468,436 (100%)	83,183 (17.76%)	385,253 (82.24%)	
*Demographic factors*					
Age at cancer diagnosis (median (range))	40008–0.0		59.90 (5.20–79.20)		
Male (*n*, %)	31–0.0	214,419 (45.77%)	35,918 (43.18%)	178,501 (46.33%)	<2.2e−16
Female (*n*, %)		254,017 (54.23%)	47,265 (56.82%)	206,752 (53.67%)	<2.2e–16
White (*n*, %)	21000–0.0	424,542 (90.73%)	77,109 (92.70%)	347,433 (90.18%)	<2.2e−16
*Clinical factors (n, %)*					
Underlying (primary) cause of death					
Diseases of the circulatory system	40001–0.0	3903 (20.80%)	819 (7.12%)	3084 (42.4%)	
Diseases of the respiratory system		1240 (6.61%)	321 (2.8%)	919 (12.6%)	
Diseases of the digestive system		728 (3.88%)	198 (1.72%)	530 (7.3%)	
Infectious and parasitic diseases		166 (0.88%)	53 (0.46%)	113 (1.6%)	
Other diseases		12,730 (67.83%)	10,105 (87.9%)	2625 (36.1%)	
Reported occurrences of cancer	40009–0.0	83,183 (17.76%)	83,183 (100%)		
Reported recurrence of cancer		20,998 (4.48%)	20,998 (25.24%)		
Histology of cancer tumour					
Epithelial	40011–0.0		70,912 (89.28%)		
Behaviour of cancer tumour					
Malignant	40012–0.0		66,057 (83.12%)		
*Genetic factors (median, range)*					
DNA concentration	22024–0.0	34.42 (10–713.84)	34.35 (10–655.87)	34.43 (10–713.84)	0.13
Affymetrix quality control metric “Cluster.CR”	22025–0.0	99.52 (97–99.92)	99.52 (97.01–99.1)	99.52 (97–99.92)	0.23
Affymetrix quality control metric “dQC”	22026–0.0	0.978 (0.88–1)	0.977 (0.89–1)	0.9784 (0.88–1)	0.12
SNP (genotypes) (*n*, %)					
rs3750965					
A A	affy5779511	211,180 (45.08%)	37,638 (45.25%)	173,542 (45.05%)	HWE = 0.65
G A		206,786 (44.14%)	36,762 (44.19%)	170,024 (44.13%)	
G G		50470 (10.77%)	8783 (10.56%)	41,687 (10.82%)	
rs35264875					
A A	affy5779776	332,599 (71.00%)	58,604 (70.45%)	273,995 (71.12%)	HWE = 0.34
T A		124,338 (26.54%)	22,512 (27.06%)	101,826 (26.43%)	
T T		11,499 (2.45%)	2067 (2.48%)	9432 (2.45%)	
rs34510004					
A A	affy5779828	1 (0.0002%)	0 (0%)	1 (0.0003%)	HWE = 0.77
A G		1179 (0.25%)	230 (0.28%)	949 (0.25%)	
G G		467,256 (99.75%)	82,953 (99.72%)	384,303 (99.75%)	
rs3829241					
A A	affy5780031	72,734 (15.53%)	13,208 (15.88%)	59,526 (15.45%)	HWE = 0
A G		218,297 (46.60%)	39,316 (47.26%)	178,981 (46.46%)	
G G		177,405 (37.87%)	30,659 (36.86%)	146,746 (38.09%)	
rs72932540					
A A	affy5782262	390,424 (83.35%)	68,628 (82.50%)	321,796 (83.53%)	HWE = 0.41
G A		74,511 (15.91%)	13,929 (16.75%)	60,582 (15.73%)	
G G		3501 (0.75%)	626 (0.75%)	2875 (0.75%)	
rs25644					
A A	affy7015041	365,875 (78.11%)	65,014 (78.16%)	300,861 (78.09%)	HWE = 0.47
G A		96,176 (20.53%)	16,990 (20.42%)	79,186 (20.55%)	
G G		6385 (1.36%)	1179 (1.42%)	5206 (1.35%)	
rs28360472					
A A	affy7015101	453,577 (96.83%)	80,430 (96.69%)	373,147 (96.86%)	HWE = 0.1
G A		14,721 (3.14%)	2724 (3.27%)	11,997 (3.11%)	
G G		138 (0.03%)	29 (0.03%)	109 (0.03%)	

### Genetic variants in *TPCN2* and *P2RX4* and the risk of developing cancer at a global level, malignant, metastatic cancer, and cancer recurrence

**Fig. 2:**
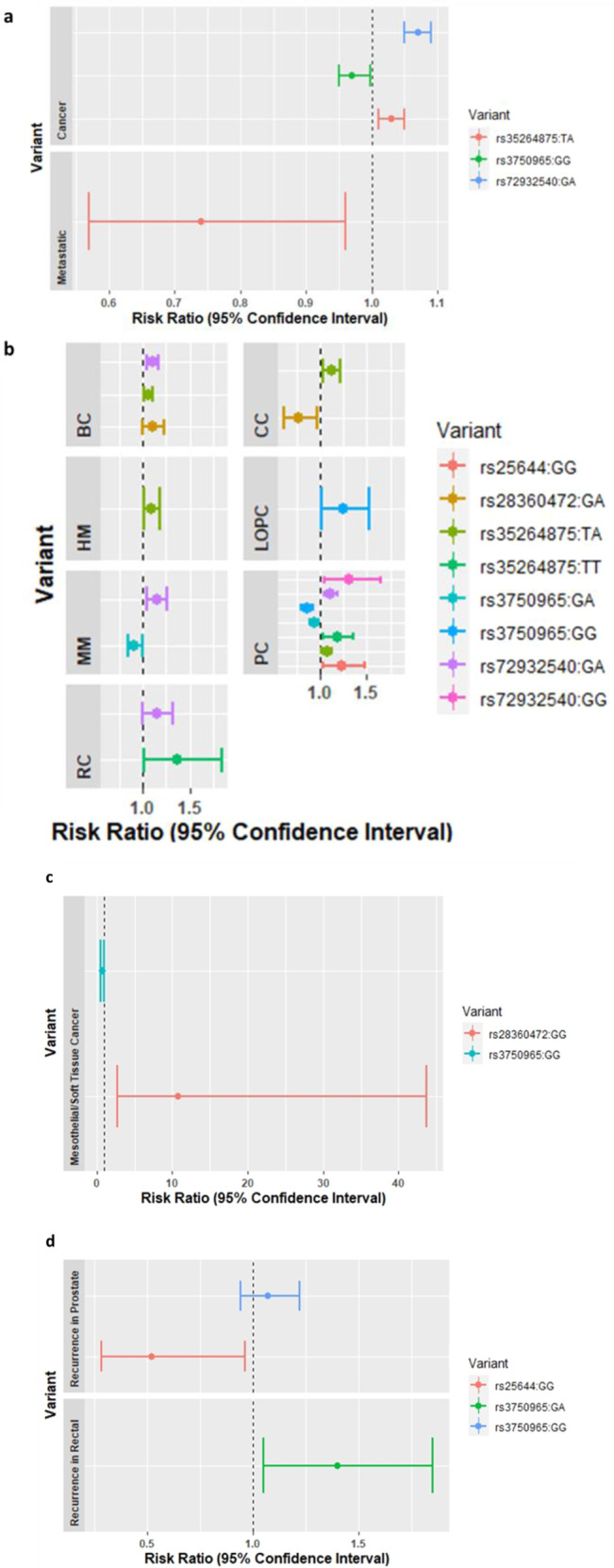
Forrest plot showing genotypic ORs for significant SNPs in *TPCN2/P2RX4*. **a**–**c** Estimates were derived using logistic regression after adjusting for sex and ethnicity for cancer at a global level or various specific types of cancer at a local level. **d** Estimates were derived using logistic regression after adjusting for age, sex, and ethnicity. BC breast cancer, HM malignant neoplasms, stated or presumed to be primary, of lymphoid, haematopoietic, and related tissue, MM malignant melanoma, PC malignant neoplasm of prostate, RC malignant neoplasm of rectum, LOPC malignant neoplasm of lip, oral cavity, and pharynx.

Carriage of *TPCN2* rs3750965:GG was associated with a lower general risk of developing cancer (odds ratio (OR): 0.97, 95% confidence interval (CI): 0.95–0.997, *P* = 0.029*, vs. A/A), whereas carriage of *TPCN2* rs35264875:TA and rs72932540:GA was associated with an increased risk of cancer susceptibility (OR: 1.03, 95% CI: 1.01–1.05, *P* = 0.001**, vs. A/A and OR: 1.07, 95% CI: 1.05–1.09, *P* = 4.51e−10***, vs. A/A, respectively) (see Table 2 and Fig. 2a). Genotype TA in rs35264875 showed lower odds of metastatic cancer (OR: 0.74, 95% CI: 0.57–0.96, *P* value = 0.025* vs. A/A; see Table 3 and Fig. 2a). No significant association was observed between these genetic variants and cancer in terms of whether it was malignant or benign and whether there was cancer recurrence (see Supplementary Tables 3 and 4).

**Table 2:** Univariate and multivariate logistic regression analyses of genetic variants in *TPCN2* and *P2RX4* that are significantly associated with the risk of cancer in the UK Biobank.

Gene	SNP	Genotype	Cancer-free (controls), *n* = 385,253	Cancer (cases), *n* = 83,183	Model A (OR, 95% CI)	Model A *P* value	Model B (OR, 95% CI)	Model B *P* value
*TPCN2*	rs3750965	GA (*n*)	170,024	36,762	1 (0.98–1.01)	0.702	1 (0.98–1.01)	0.591
		GG (*n*)	41,687	8783	0.97 (0.95–0.997)	0.026*	0.97 (0.95–0.997)	0.029*
	rs35264875	TA (*n*)	101,826	22,512	1.03 (1.02–1.05)	0.0001***	1.03 (1.01–1.05)	0.001**
		TT (*n*)	9432	2067	1.03 (0.98–1.08)	0.325	1.022 (0.97–1.07)	0.374
Close to *TPCN2*	rs72932540	GA (*n*)	13,929	60,582	1.08 (1.06–1.10)	2.76e–13***	1.07 (1.05-1.09)	4.51e−10***
		GG (*n*)	626	2875	1.02 (0.94–1.113)	0.639	1.012 (0.93–1.103)	0.793
*P2RX4*	rs28360472	GA (*n*)	2724	11,997	1.053 (1–1.099)	0.0159*	1.04 (0.99–1.08)	0.091
		GG (*n*)	29	109	1.23 (0.82–1.9)	0.314	1.23 (0.81–1.85)	0.331

Model A: univariate logistic regression; model B: multivariate logistic regression, adjusted for sex and ethnicity.

*OR* odds ratio, *CI* confidence interval.

*p < 0.05 **p ≤ 0.005 ***p ≤ 0.0005.

**Table 3:** Univariate and multivariate logistic regression analyses of a genetic variant (rs35264875) that significantly associated with risk of metastatic cancer.

Gene	SNP	Genotype	Primary cancer (controls), *n* = 65,559	Metastatic cancer (cases), *n* = 334	Model A (OR, 95% CI)	Model A *P* value	Model B (OR, 95% CI)	Model B *P* value	Model C (OR, 95% CI)	Model C *P* value
*TPCN2*	rs35264875	TA (*n*)	17,864	73	0.74 (0.57–0.96)	0.024*	0.74 (0.57–0.96)	0.0235*	0.74 (0.57–0.96)	0.025*
		TT (*n*)	1654	7	0.77 (0.36–1.6)	0.49	0.77 (0.36–1.63)	0.4911	0.77 (0.36–1.63)	0.49

Model A: univariate logistic regression; model B: multivariate logistic regression, adjusted for sex and ethnicity; model C: multivariate logistic regression, adjusted for age, sex, and ethnicity.

*OR* odds ratio, *CI* confidence interval.

*p < 0.05.

### Genetic variants in *TPCN2* and *P2RX4* and the risk of developing various types of cancer at a local level

Carriage of *TPCN2* rs35264875:TA or rs72932540:GA or *P2RX4* rs28360472:GA was associated with a higher general risk of developing breast cancer (OR: 1.06, 95% CI: 1.02–1.1, *P* = 0.006**, OR: 1.1, 95% CI: 1.05–1.16, *P* = 7.2e−05***, OR: 1.1, 95% CI: 1–1.22, *P* = 0.0478*, respectively, vs. A/A). Carriage of *TPCN2* rs35264875: TA was associated with an increased risk of colon cancer susceptibility (OR: 1.03, 95% CI: 1.01–1.05, *P* = 0.00138**, vs. A/A), whereas carriage of *P2RX4* rs28360472:GA was associated with a decreased risk of colon cancer susceptibility (OR: 0.77, 95% CI: 0.61–0.97, *P* = 0.028* vs. A/A) (see Table 4 and Fig. 2b). Genotype TA in rs35264875 showed higher odds of malignant neoplasms, stated or presumed to be primary, of lymphoid, haematopoietic, and related tissue (OR: 1.09, 95% CI: 1.02–1.18, *P* value = 0.015* vs. A/A) (see Table 4 and Fig. 2b). Genotype GA in rs72932540 (in close proximity to the *TPCN2* gene) showed higher odds of malignant melanoma (MM) (OR: 1.15, 95% CI: 1.042–1.26, *P* value = 0.00512** vs. A/A), whereas genotype GA in rs3750965 in *TPCN2* showed lower odds of MM (OR: 0.91, 95% CI: 0.84–0.99, *P* value = 0.02334* vs. A/A) (see Table 4 and Fig. 2b). Carriage of *TPCN2* rs3750965:GG was associated with a lower risk of developing malignant neoplasms of mesothelial and soft tissue (MS) (OR: 0.72, 95% CI: 0.53–0.97, *P* = 0.032*, vs. A/A); in contrast, it was associated with an increased risk of malignant neoplasms of the lip, oral cavity, and pharynx (OR: 1.25, 95% CI: 1.02–1.53, *P* = 0.0311* vs. A/A). Carriage of *P2RX4* rs28360472:GG was associated with an increased risk of MS susceptibility (OR: 10.8, 95% CI: 2.7–43.7, *P* = 0.0009***, vs. A/A; see Table 4 and Fig. 2c). Genotypes GA/GG in *TPCN2* rs3750965 showed lower odds of malignant neoplasms of the prostate (OR: 0.94, 95% CI: 0.90–0.99, *P* = 0.017*, OR: 0.86, 95% CI: 0.80–0.93, *P* = 0.0002***, respectively, vs. A/A), whereas genotypes TA/TT in rs35264875 or GA/GG in rs72932540 in *TPCN2* or genotype GG in rs25644 in *P2RX4* showed higher odds of malignant neoplasms of the prostate (OR: 1.073, 95% CI: 1.02–1.13, *P* = 0.005**, OR: 1.18, 95% CI: 1.03–1.35, *P* = 0.016*, OR: 1.11, 95% CI: 1.04–1.18, *P* = 0.0007***, OR: 1.31, 95% CI: 1.04–1.65, *P* = 0.024*, OR: 1.23, 95% CI: 1.03–1.47, *P* = 0.02175*, respectively, vs. A/A) (see Table 4 and Fig. 2b). Carriage of *TPCN2* rs35264875:TT or rs72932540:GA was associated with a higher risk of developing malignant neoplasm of the rectum (OR: 1.37, 95% CI: 1.02–1.84, *P* = 0.034*, OR: 1.15, 95% CI: 1.004–1.32, *P* = 0.044*, respectively, vs. A/A). No significant association was observed between these genetic variants and the risk of developing malignant neoplasms of the brain, bronchus, lungs, ovaries, thyroid gland, or urinary tract (see Supplementary Table 5).

**Table 4:** Univariate and multivariate logistic regression analyses of genetic variants in *TPCN2* and *P2RX4* that significantly associated with the risk of various types of cancer at a local level.

Gene	SNP	Genotype	Cancer-free (controls), *N* = 385,253	Cancer subtype (cases)	Cancer subtype	Cancer subtype cohort sample size (*n*)	Model A (OR, 95% CI)	Model A *P* value	Model B (OR, 95% CI)	Model B *P* value
*TPCN2*	rs3750965	GA (*n*)	170,024	381	LOPC	918	0.94 (0.82–1.08)	0.3975	0.94 (0.82–1.08)	0.3839
				3494	PC	8025	0.95 (0.91–0.996)	0.035*	0.94 (0.90–0.99)	0.017*
				299	MS	632	1.07 (0.91–1.3)	0.386	1.07 (0.91–1.3)	0.385
				1204	MM	2836	0.915 (0.85–0.99)	0.027*	0.91 (0.84–0.99)	0.023*
		GG (*n*)	41,687	124	LOPC	918	1.25 (1.02–1.53)	0.0296*	1.25 (1.02–1.53)	0.0311*
				290	MM	2836	0.9 (0.79–1)	0.103	0.9 (0.8–1.02)	0.106
				781	PC	8025	0.87 (0.80–0.94)	0.0003***	0.86 (0.80–0.93)	0.0002***
				49	MS	632	0.72 (0.53–0.97)	0.033*	0.72 (0.53–0.97)	0.032*
	rs35264875	TA (*n*)	101,826	385	RC	1426	1.04 (0.93–1.17)	0.492	1.03 (0.92–1.16)	0.575
				2240	PC	8025	1.085 (1.03–1.14)	0.001**	1.073 (1.02–1.13)	0.005**
				1038	HM	3666	1.1 (1.02–1.18)	0.012*	1.09 (1.02–1.18)	0.015*
				859	CC	2996	1.12 (1.03–1.21)	0.005**	1.12 (1.03–1.21)	0.006**
				3322	BC	12,056	1.06 (1.02–1.1)	0.00578**	1.06 (1.02–1.1)	0.006**
		TT (*n*)	9432	47	RC	1426	1.37 (1.02–1.84)	0.034*	1.37 (1.02–1.84)	0.034*
				228	PC	8025	1.19 (1.04–1.36)	0.01*	1.18 (1.03–1.35)	0.016*
				83	HM	3666	0.95 (0.76–1.18)	0.630	0.95 (0.76–1.18)	0.623
				75	CC	2996	1.06 (0.84–1.33)	0.641	1.06 (0.84–1.34)	0.625
				294	BC	12,056	1.012 (0.9–1.14)	0.84415	1.01 (0.9–1.14)	0.821
Close to *TPCN2*	rs72932540	GA (*n*)	60,582	255	RC	1426	1.12 (1.02–1.34)	0.025*	1.15 (1.004–1.32)	0.044*
				1390	PC	8025	1.13 (1.06–1.19)	7.34e−05***	1.11 (1.04–1.18)	0.0007***
				508	MM	2836	1.17 (1.06–1.3)	0.002**	1.15 (1.042–1.26)	0.005**
				2063	BC	12,056	1.11 (1.05–1.16)	3.67e−05***	1.1 (1.05–1.16)	7.2e−05***
				12	RC	1426	1.16 (0.7–2.05)	0.6121	1.16 (0.7–2.05)	0.6100
		GG (*n*)	2875	76	PC	8025	1.3 (1.03–1.63)	0.0261*	1.31 (1.04–1.65)	0.024*
				93	BC	12,056	1.05 (0.85–1.29)	0.635	1.03 (0.84–1.27)	0.7679
				19	MM	2836	0.92 (0.59–1.45)	0.722	0.91 (0.58–1.42)	0.667
*P2RX4*	rs25644	GA (*n*)	79,186	1648	PC	8025	1.002 (0.95–1.06)	0.9297	1.008 (0.95–1.07)	0.79076
				538	MM	2836	0.91 (0.83–1)	0.0423*	0.92 (0.83–1.01)	0.073
		GG (*n*)	5206	44	MM	2836	1.13 (0.84–1.52)	0.4303	1.16 (0.86–1.56)	0.33
				131	PC	8025	1.21 (1.02–1.44)	0.0314*	1.23 (1.03–1.47)	0.02175*
	rs28360472	GA (*n*)	11,997	19	MS	632	0.97 (0.61-1.53)	0.886	0.96 (0.61-1.52)	0.864
				73	CC	2996	0.78 (0.62–0.98)	0.034*	0.77 (0.61–0.97)	0.028*
				418	BC	12,056	1.12 (1.01–1.23)	0.0283*	1.11 (1–1.22)	0.0478*
		GG (*n*)	109	2	MS	632	11.21 (2.8–45.48)	0.0007***	10.8 (2.7–43.7)	0.0009***
				1	CC	2996	1.17 (0.16–8.4)	0.875	1.14 (0.16–8.2)	0.897
				3	BC	12,056	0.88 (0.280–2.78)	0.8312	1.02 (0.32–3.27)	0.9737

Model A: univariate logistic regression; model B multivariate logistic regression, adjusted for sex and ethnicity.

*OR* odds ratio, *CI* confidence interval, *BC* breast cancer, *HM* malignant neoplasms, stated or presumed to be primary, of lymphoid, haematopoietic, and related tissue, *MM* malignant melanoma, *MS* malignant neoplasms of mesothelial and soft tissue, *LOPC* malignant neoplasm of the lip, oral cavity, and pharynx, *PC* malignant neoplasm of the prostate, *RC* malignant neoplasm of the rectum.

*p < 0.05 **p ≤ 0.006 ***p ≤ 0.0009.

### Genetic variants in *TPCN2* and *P2RX4* and cancer recurrence in patients with different types of cancer

Carriage of *TPCN2* rs3750965:GG was associated with an increased risk of cancer recurrence susceptibility in patients with prostate cancer (OR: 1.07, 95% CI: 0.94–1.22, *P* = 0.018* vs. A/A), whereas carriage of *P2RX4* rs25644:GG was associated with a decreased risk of cancer recurrence susceptibility (OR: 0.52, 95% CI: 0.28–0.96, *P* = 0.038* vs. A/A), after adjusting for age as well as sex and ethnicity (see Table 5 and Fig. 2d). Genotype GA in rs3750965 showed higher odds of cancer recurrence in patients with rectal cancer after adjusting for age as well as sex and ethnicity (OR: 1.4, 95% CI: 1.05–1.85, *P* value = 0.0199* vs. A/A), as shown in Table 5 and Fig. 2d. No significant association was observed between these genetic variants and the risk of cancer recurrence in patients with malignant neoplasms of the brain, breast, bronchus, lungs, and colon and malignant neoplasms, stated or presumed to be primary, of the lymphoid, haematopoietic and related tissue, melanoma, MS, ovaries, thyroid gland, or urinary tract (see Supplementary Table 6).

**Table 5:** Univariate and multivariate logistic regression analyses of genetic variants in *TPCN2* and *P2RX4* that significantly associated with the risk of cancer recurrence in the UK Biobank.

Gene	SNP	Genotype	Cancer occurrence once (controls), *n*	Cancer recurrence (cases), *n*	Cancer subtype	Cancer cohort (*n*)	Model A (OR, 95% CI)	Model A *P* value	Model B (OR, 95% CI)	Model B *P* value	Model C (OR, 95% CI)	Model C *P* value
*TPCN2*	rs3750965	G A	525	2969	PC	8025	1.08 (0.94–1.23)	0.268	1.07 (0.94-1.22)	0.294	1.29 (1.04–1.58)	0.312
			141	483	RC	1426	1.36 (1.03 –1.8)	0.028*	1.37 (1.04–1.81)	0.026*	1.4 (1.05–1.85)	0.0199*
		G G	135	646	PC	8025	1.27 (0.94–1.57)	0.023*	1.28 (1.04–1.57)	0.020*	1.07 (0.94–1.22)	0.018*
			27	134	RC	1426	0.94 (0.6–1.5)	0.798	0.97 (0.61–1.54)	0.887	1.01 (0.63–1.6)	0.981
*P2RX4*	rs25644	G A	240	1408	PC	8025	0.96 (0.83–1.12)	0.645	0.96 (0.82–1.12)	0.589	0.95 (0.82–1.11)	0.5482
		G G	11	120	PC	8025	0.52 (0.28–0.97)	0.038*	0.52 (0.28–0.96)	0.038*	0.52 (0.28–0.96)	0.038*

Model A: univariate logistic regression; model B multivariate logistic regression, adjusted for sex and ethnicity; model C multivariate logistic regression, adjusted for age, sex, and ethnicity.

*OR* odds ratio, *CI* confidence interval, *PC* malignant neoplasm of the prostate, *RC* malignant neoplasm of the rectum.

*p < 0.05.

### Association between significant single-nucleotide polymorphisms (SNPs) and *TPCN2*/*PR2X4* expression/activity levels

The association between our significant variants and their predicted effect on RNA/protein levels in a similar biological/clinical context is summarised in Table 6.

**Table 6:** Predictions of the association between significant SNPs and TPCN2/PR2X4 expression/activity levels using bioinformatics tools.

Gene	SNP	Genotype	Example of a significant association in our study	eQTL analysis using data in GTEx database (fold change of median genotype compared to median WT)	Pathogenicity predication			Predicted potential impact on protein (AA variant) (Mutation Assessor release 3)	Protein expression levels in this type of cancer (Human Protein Atlas)	Published evidence
					(CADD GRCh38-v1.6)		(FATHMM-XF)			
					PHRED	Raw Score	Score			
*TPCN2*	rs3750965	GG	Decreased risk of MM	Decrease in *TPCN2* expression by 8.76-fold in GG compared to AA in the skin—not sun-exposed (Suprapubic)	6.6	0.51	0.07	Medium (K376R)	Medium/high expression of protein was detected in 3 out of 11 patients with MM	NA
	rs35264875	TA/TT	Increased risk of PC	Increase in *TPCN2* expression by 1.76 in TA compared to AA in the prostate	9.81	0.84	0.18	Low (M484L)	Medium/high expression of protein was detected in 10 out of 11 patients with PC	Gain of TPC2 function (ref. ^29^)
*P2RX4*	rs25644	GG	Increased risk of PC	Increase in *P2RX4* expression by 3.8 in GA compared to AA in prostate. Not enough data to estimate expression in GG compared to AA in prostate. However, we have investigated this in a different tissue (adipose—subcutaneous) and found an incremental difference in the expression by 4.79 in GG compared to AA	23.5	3.04	0.28	Medium (S242G)	Medium/high expression of protein was detected in12 out of 12 patients with PC	NA
	rs28360472	GA/GG	Increased risk of MS	NA	26.1	3.86	0.69 (pathogenic)	Medium (Y315C)	NA	Loss of function of P2RX4 (ref. ^40^)

Main sources: https://www.gtexportal.org; https://cadd.gs.washington.edu/snv; FATHMM-XF—predicts the functional consequences of single-nucleotide variants (SNVs) with extra features (biocompute.org.uk); http://mutationassessor.org/r3/; The Human Protein Atlas.

*eQTL* expression quantitative trait loci, *GTEx* Genotype-Tissue Expression Project database, *HM* malignant neoplasms (stated or presumed to be primary of lymphoid, haematopoietic, and related tissue), *MM* malignant melanoma, *MS* malignant neoplasms of mesothelial and soft tissue, *PC* malignant neoplasm of the prostate.

## Discussion

Cancer is a disease with both genetic and environmental contributors accounting for substantial morbidity and mortality rates on a global scale. Cancer genomics is an evolving scientific discipline that applies human genetics to ensure that the best and most tailored therapy is provided to each cancer patient in clinical settings. Over recent years, the number of Food and Drug Administration-approved drugs based on genetic information in oncology has been rising rapidly and this has allowed important advances in the treatment of cancer to be made, giving many patients hope for a cure. Genetics provides a clinical tool to stratify patients and an approach to aid the development of novel, safer, and more efficacious antineoplastic agents. Although endolysosomal ion channels have been shown to play roles in pathophysiological processes related to cancer^1,18^, a paucity of clinical investigations have been conducted on cancer patients to provide genetic evidence to support the experimental data. In this study, we leveraged a large, single resource, the UK Biobank, to explore for the first time the relationship between genetic variants of two endolysosomal proteins, *TPCN2* and *P2RX4*, and human cancer. This is the first human genetic study to identify risk variants in endolysosomal ion channels that contribute to cancer at the global level as well as in 13 specific subsets of cancer in terms of cancer risk, disease recurrence, malignancy, and metastasis in the UK Biobank’s population.

The molecular mechanisms that underpin the role of endolysosomal ion channels, particularly TPC2, in various fundamental processes of oncogenesis, in different types of cancer, and at different stages of tumour development are gradually being unravelled^19–21^. TPC2 and P2X4 are endolysosomal cation channels located in the endolysosomal system and their genes are found on chromosome 11, region 13.2, and chr12, region 24.31, respectively^22,23^. Data from several sources associate 11q13-q14 amplifications with cancer^24^. We identified polymorphisms in the *TPC2N* and *P2RX4* genes that are significantly associated with both an increased and decreased risk for developing cancer or a subtype of cancer in the UK Biobank. This study focusses on the endolysosomal ion channels, TPC2 and P2RX4, for several reasons. There is increasing evidence linking TPC2 to cancer—the *TPC2N* gene is located on chromosome 11, region 13.2, a genomic region amplification of which has been found to be correlated with cancer^22,24,25^. Our understanding of purinergic receptor P2X4’s role in cancer is evolving; it is a player in physiological processes related to tumour growth involving proliferation and apoptosis, which are two of the hallmarks of cancer^12^. Cationic amphiphilic drug (CAD) repurposing is an emerging therapeutic approach for cancer therapy, and recently a study demonstrated that the CAD/P2XR4/ADCY1/Ca^2+^ signalling pathway is critical for CAD-induced lysosome-dependent cell death^26^, although the cellular mechanisms underlying this phenomenon are a matter of speculation. In addition, it has been shown that increased expression of the P2X4 receptor is significantly correlated in Pakistani patients with hepatocellular carcinoma, adenocarcinoma, and ampullary carcinoma^27^. rs35264875 in *TPCN2* is a non-synonymous, coding, and missense genetic variant that has previously been associated with a shift from brown to blond hair^28^. It leads to a non-synonymous substitution of methionine to leucine and was functionally characterised by endolysosomal patch-clamp techniques, which demonstrated that this genetic variant leads to a gain of TPC2 function via conformational changes within the pore^29^. We found that the carrying of TPCN2 rs35264875:TA was associated with an increased risk of cancer susceptibility (OR: 1.03, 95% CI: 1.01–1.05, *P* = 0.00138**, vs. A/A) and a decreased risk of cancer metastasis (OR: 0.74, 95% CI: 0.57–0.96, *P* value = 0.025* vs. A/A). Carrying of rs35264875:TA was associated with a higher risk of developing breast and colon cancer and malignant neoplasms, stated or presumed, to be primary of lymphoid, haematopoietic, and related tissue, as well as cancer of the prostate or rectum (OR: 1.06, 95% CI: 1.02–1.1, *P* = 0.006**, OR: 1.03, 95% CI: 1.01–1.05, *P* = 0.00138**, OR: 1.09, 95% CI: 1.02–1.18, *P* value = 0.015*, OR: 1.073, 95% CI: 1.02–1.13, *P* = 0.005**, OR: 1.37, 95% CI: 1.02–1.84, *P* = 0.034*, respectively, vs. A/A). These findings are consistent with previous studies that identified TPCN2 overexpression as a potential risk factor for skin cancer^30^. We navigated Genotype-Tissue Expression (GTEx) to determine the effect of heterozygote rs35264875 in *TPCN2* expression in the prostate, and we found that *TPCN2* expression was upregulated (see Table 6) in TCGA. *TPCN2* expression was significantly downregulated in primary compared to metastatic patients with human skin cutaneous melanoma^8^. In addition to the reported reduction in *TPCN2* expression in melanoma by D’Amore et al.^8^, we found that there is a significantly decreased pattern of *TPCN2* expression associated with this cancer stage in patients with uveal melanoma (expression of TPCN2 in patient with uveal melanoma, stratified by cancer stage (1 vs. 2, *P* value, 3.203800e−01, 2 vs. 3, *P* value 1.834220e−01, 3 vs. 4, *P* value <1e−12), using the UALCAN database^31^. These data are in accordance with our findings that heterozygote rs35264875 is associated with an increased risk of cancer and a decreased risk of cancer metastasis indicating that *TPCN2* overexpression acts as a driver of tumorigenesis and *TPCN2* reduced expression acts as an enhancer of metastatic phenotypes. Expression of this *TPCN2* variant was found to be significantly associated with increased survival in bladder cancer (*P* value = 3.56e−02)^32^. This variant is common among Europeans (minor allele frequency (MAF) > 15%)^33^, suggesting that rs35264875 represents an important genetic biomarker in the definition of tumour types (including cancers of the breast or colon, haematological malignancies, prostate, and rectal), susceptibility, and metastasis. Our findings provide genetic evidence that TPC2 behaves differently in various stages/types of cancer and indicates that TPC2 gain of function can contribute to tumorigenesis but may hinder metastasis, which warrants further investigations at a molecular level.

rs3750965 in *TPCN2* is a missense variant^34^; it is a coding sequence variant that substitutes A (AAA) in the amino acid codon with G (AGA). This was predicted using Ensembl Variant Effect Predictor (VEP) and it leads to the amino acid lysine being substituted by arginine. The effect of this genetic variant on TPC2 protein structure or function has not yet been established. We observed that carriage of *TPCN2* rs3750965:GG was associated with a lower risk of developing cancer at a global level (OR: 0.97, 95% CI: 0.95–0.997, *P* = 0.0292*, vs. A/A) and genotypes GA/GG with a significantly decreased risk of developing MM, MS, and malignant neoplasm of the prostate (GA OR: 0.91, 95% CI: 0.84–0.99, *P* value = 0.02334*, GA OR: 0.72, 95% CI: 0.53–0.97, *P* = 0.032*, GA OR: 0.94, 95% CI: 0.90–0.99, *P* = 0.017*, GG OR: 0.86, 95% CI: 0.80–0.93, *P* = 0.0002***, respectively, vs. A/A), suggesting that rs3750965 is a protective genetic marker against cancer at a global level and various subtypes of cancer, including melanoma, mesothelial and soft tissue cancer, and prostate cancer. We detected downregulation of *TPCN2* expression in skin that is not sun-exposed (suprapubic) in carriers of the homozygous variant rs3750965 in GTEx. We speculate that there exists an association of this genetic variant with a decreased risk of cancer at a global level, and MM and MS at a local level, due to a decrease in *TPCN2* expression that has been shown to be functionally expressed in different types of cancer, using the Human Protein Atlas. Huang et al. observed that TPC2 is overexpressed in oral squamous cell carcinoma cell lines^25^. In our study, we identified a significant association of the GG genotype in rs3750965 with a higher risk of malignant neoplasms of the lip, oral cavity, and pharynx, and we are not able to speculate about the effect of this genotype on channel expression/activity in human oral tissues because of lack of data. We believe that TPC2 has distinct roles in various processes, stages, and types of cancers. We also found that carriage of *TPCN2* rs3750965:GG/GA is associated with an increased risk of cancer recurrence susceptibility in patients with prostate cancer and rectal cancer (GG OR: 1.07, 95% CI: 0.94–1.22, *P* = 0.018, GA OR: 1.4, 95% CI: 1.05–1.85, *P* value = 0.0199*, respectively, vs. A/A), indicating the possibility of utilising this genetic variant to predict cancer recurrence in patients with prostate and rectal cancer. rs72932540 is a genetic variant located upstream of the *TPCN2* TSS on GRCh38: 11:69,154,575. The distance from the variant to the TSS of *TPCN2* is 105,643 Kb^9,35^. This genetic variation at close proximity to *TPCN2* might influence the expression levels of TPC2 and thereby tumour development. We found that rs72932540:GA was associated with an increased general risk of cancer susceptibility (OR: 1.07, 95% CI: 1.05–1.09, *P* = 4.51e−10***, vs. A/A) and a higher risk of developing breast cancer, MM, and rectal cancer (OR: 1.1, 95% CI: 1.05–1.16, *P* = 7.2e−05***, OR: 1.15, 95% CI: 1.042–1.26, *P* value = 0.00512**, OR: 1.15, 95% CI: 1.004–1.32, *P* = 0.044*, respectively, vs. A/A). Carriage of genotypes GA/GG in rs72932540 was associated with higher odds of malignant neoplasm of the prostate (GA OR: 1.073, 95% CI: 1.02–1.13, *P* = 0.005**, GG OR: 1.18, 95% CI: 1.03–1.35, *P* = 0.016*), raising a question regarding the clinical utility of rs72932540 as a diagnostic genetic biomarker for cancer or subtypes of cancer, such as breast cancer, MM, and cancers of the prostate and rectum.

Our data regarding rs72932540 in the context of breast cancer is consistent with a genome-wide association study (GWAS) that discovered this genetic variant’s (*P* value 4 × 10^−8^) correlation with an increased risk of breast cancer in the European population^10^. Causal factors leading to breast cancer remain a matter of speculation; established risk genetic factors are required to stratify individuals who are more likely to develop breast cancer, which may have significant implications for their care. Our findings confirmed GWAS data, which provides additional evidence that rs72932540 is a potential genetic risk marker for breast cancer; how it influences the development of cancer is a question for future research. There is a growing body of literature that recognises the importance of *TPCN2* in prostate cancer. It is one among six gene signatures associated with prostate cancer to predict postoperative biochemical recurrence^36^. We identified two genetic variants in *TPCN2* that are significantly associated with prostate cancer susceptibility and disease recurrence in patients with prostate cancer, which necessitates investigating the causal mechanisms underlying the role of these genetic variants in prostate cancer development. Previous studies demonstrated that P2RX4 acts as a regulator of tumour development by playing a critical role in inflammation and immune cell function, which are part of the pathophysiological processes that occur in the tumour microenvironment^37,38^. rs25644 (Ser242>Gly) and rs28360472 (Tyr315>Cys) are nonsynonymous coding SNPs in the *P2RX4* gene^39^. We found that rs25644:GG in *P2RX4* is associated with increased risk of prostate cancer (OR: 1.23, 95% CI: 1.03–1.47, *P* = 0.02175* vs. A/A) but with a decreased risk of cancer recurrence in patients with prostate cancer (OR: 0.52, 95% CI: 0.28–0.96, *P* = 0.038*, vs. A/A). He et al. revealed the pathophysiological significance of P2RX4 in prostate tumorigenesis. They found that there is a disruption of P2RX4 function by 5-(3-bromophenyl)-1,3-dihydro-2Hbenzofuro [3,2-e]-1,4-diazepin-2-one diminished prostate cancer growth in vitro and in vivo^17^. They also found a significant correlation between P2RX4 overexpression and prostate cancer^17^. Using bioinformatics tools (GTEx and UALCAN), we found that the homozygous genetic variant rs25644 contributed to an elevated expression of P2RX4 in the prostate, and P2RX4 was significantly upregulated in patients with prostate adenocarcinoma compared to healthy subjects (*P* value, 2.23310259173104e−12)^31^. This explains our finding that a genetic variant is significantly correlated with an increased risk of prostate cancer and provides insights into the role that P2RX4 plays in prostate cancer pathology. Many unanswered questions remain about the role of P2RX4 in prostate tumorigenesis at the molecular level, warranting the functional characterisation of *P2RX4* polymorphisms in prostate cancer with potential translational relevance. While there is a paucity of data on the effect of this genetic variant on P2RX4 function, this candidate genetic marker in prostate cancer development warrants further independent replication or functional validation of this genetic variant. A previous study investigated the influence of rs28360472 on P2RX4 and found that it leads to a loss of function of the P2RX4 receptor^40^. rs28360472:GA/GG was associated with a higher risk of developing breast cancer and of MS susceptibility (GA OR: 1.1, 95% CI: 1–1.22, *P* = 0.0478*, GG OR: 10.8, 95% CI: 2.7–43.7, *P* = 0.0009***, vs. A/A). In contrast, it was associated with a decreased risk of colon cancer susceptibility (OR: 0.77, 95% CI: 0.61–0.97, *P* = 0.028* vs. A/A). rs28360472 was shown to lead to loss of function of P2RX4 by disrupting the agonist binding site^40^, which suggests that this change in P2RX4 function plays a role in increasing susceptibility to breast cancer and of MS susceptibility, while reducing susceptibility to colon cancer. We reported the predicted pathogenicity scores of CADD GRCh38-v1.6 and FATHMM-XF (26.1 and 0.69, respectively), which means that a pathogenicity was likely being shown to be associated with a loss of function of P2RX4. We observed significant reduction of *P2RX4* expression in patients with mesothelioma stratified by presence/absence of a TP53 mutation (*P* value, 3.569600e−01) using the UALCAN database^31^ and infer that the increase of mesothelioma risk associated with this genetic mutation can be attributed to the reduction in P2RX4 activity. P2RX4 might demonstrate distinctive roles in different types of cancer. These genetic findings highlight, for the first time, the involvement of P2RX4 endolysosomal ion channels in various aspects of cancer phenotypes other than prostate cancer growth, cancer pain, and tumour microenvironment. The data from logistic regression were adjusted for age, sex, and ethnicity, which are significant confounders and risk factors for cancer. The key strength of this study that it is the first well-characterised genetic association study performed on a large population to discover genetic variants in genes coding for endolysosomal ion channels that correlate with cancer at a global level and in 13 subtypes of cancer in terms of cancer risk, disease recurrence, malignancy, and metastasis. Adjustments were not made for cancer recurrence for two possible confounders, chemotherapy treatment and radiation therapy, which is a limitation of this study. Another limitation is that it is retrospectively conducted on individuals, mainly Europeans; hence, our findings are not necessarily inferable and generalisable to other populations. We have observed several significant associations between heterozygous genetic variants and cancer/cancer outcomes but no significant associations for the corresponding homozygous genotypes of these variants; we speculate that these observations are due to higher frequency of the heterozygous genotypes of these variants in the case groups compared to the control groups, and we eliminate the possibility of the sample size being sufficient to detect the significant difference in the heterozygous groups due to similarity between heterozygosity case/control ratios and homozygosity case/control ratios. Further studies are required to validate these findings and provide scientific insights into these observations as the genomics field of endolysosomal cation channels (especially TPC2 and P2RX4) is evolving. Beyond the associations of these genetic variants with cancer/cancer subtype phenotypes, we have predicted the possible biological involvement of these polymorphisms in cancer/cancer subtype traits. While these predictions and speculations are intriguing, functional characterisation of these novel associations is required to develop a full picture of the contribution of these significant variants to increased/decreased risk of cancer/cancer subtype phenotypes. In summary, our study found a novel association between genetic variants in *TPCN2* and *P2RX4* and the risk of developing cancer, metastatic cancer, cancer recurrence at a global level, or various types of cancer at the local level in the UK Biobank population. This study lays the groundwork for future research to validate our findings in prospective cohorts with diverse populations and also for a functional analysis of significant genetic variants to reveal whether these associations reflect causal roles in cancer phenotypes and the exploration of their clinical utility. Future study of these genetic variants could contribute to the identification of novel cancer biomarkers and aid development of new diagnostic and therapeutic strategies, representing a further step towards personalised cancer care.

## Methods

### Study participants

**Fig. 3:**
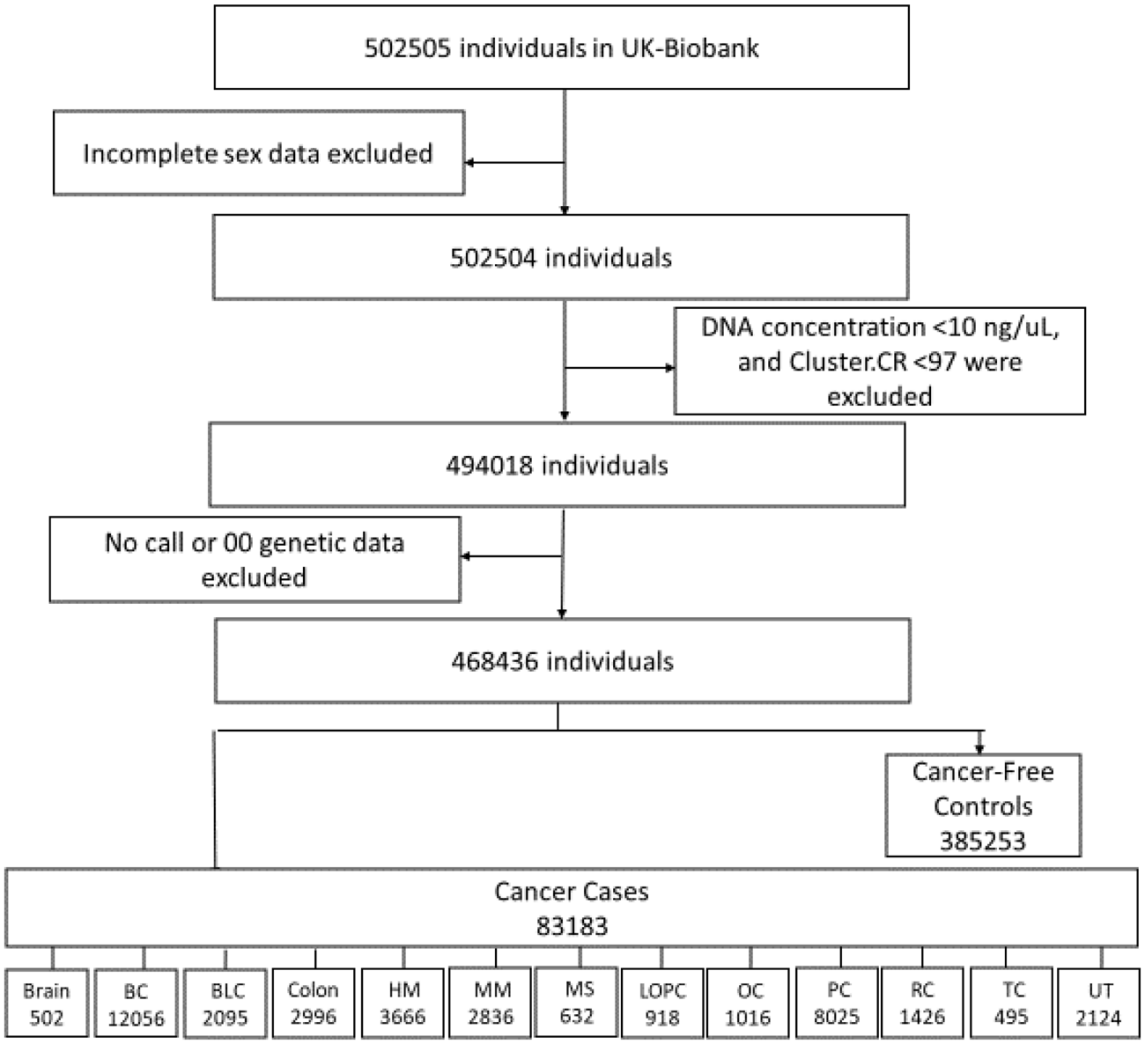
Sample selection strategy. BC breast cancer, BLC bronchus and lung cancer, HM malignant neoplasms, stated or presumed to be primary, of lymphoid, haematopoietic and related tissue, MM malignant melanoma, MS malignant neoplasms of mesothelial and soft tissue, LOPC malignant neoplasm of the lip, oral cavity, and pharynx, OC malignant neoplasm of the ovary, PC malignant neoplasm of the prostate, RC malignant neoplasm of the rectum, TC malignant neoplasm of the thyroid gland, UT malignant neoplasm of the urinary tract.

We leveraged the UK Biobank’s large-scale data, which includes >500,000 clinically and genetically assessed participants, to conduct a case–control study. Our study population consisted of 2 major cohorts and 13 sub-cohorts defined as cases and controls. Participants with complete sex and genetic data, DNA concentration ≥10 ng/μL, and cluster CR ≥ 97 were eligible (see Fig. 3). We defined metastasis and malignancy by behaviour of cancer tumour data (40012-0.0), cancer recurrence by reported occurrences of cancer (≥2 [40009-0.0]), and the cancer type by type of cancer ICD-10 (40006-0.0), which was originally obtained from the cancer register.

### DNA and genotyping

Genomic DNA was extracted from the participants and stored according to the UK Biobank’s procedures. Genotyping of the SNPs was performed using the GeneTitan^®^ Multi-Channel Instrument at Affymetrix Research Services Laboratory in Santa Clara, CA, USA, based on the UK Biobank’s protocols.

### Genetic analysis

We investigated the associations between SNPs in *TPCN2* (rs3750965, rs35264875, rs34510004, and rs3829241), a variant in close proximity to the *TPCN2* gene (rs72932540), and *P2RX4* (rs25644 and rs28360472) and cancer in the UK Biobank data. The MAFs were 33, 16, 0.13, 39, 9, 12, and 2% for rs3750965, rs35264875, rs34510004, rs3829241, rs72932540, rs25644, and rs28360472, respectively, and were all in Hardy–Weinberg equilibrium (*P* value > 0.05)—except for rs3829241 (*P* value < 0.05), which was excluded from the analysis (see Supplementary Table 7). Genetic associations with the risks of developing cancer at the global level or various types of cancer at the local level, of developing malignant or metastatic tumours, and of cancer recurrence were determined using univariate and multivariate logistic regression analyses after adjusting for significant cofounders. We first assessed the genetic association risk among cases and controls and then we investigated the significant values using logistic regression analysis. The associations were assessed using three logistic regression models: Model A: univariate logistic regression; model B: multivariate logistic regression after adjusting for sex and ethnicity, and model C: multivariate logistic regression after adjusting for age (as a categorical variable), sex, and ethnicity.

### Bioinformatics analyses

We navigated several public databases and tools (including the GTEx^41^, Combined Annotation Dependent Depletion (CADD) GRCh38-v1.6^42^, FATHMM-XF^43^, MutationAssessor release 3^44^, Human Protein Atlas^45^, Ensembl VEP^34^, and UALCAN^31^) to predict the possible effect of the significant genetic variants in *TPCN2*/*PR2X4* expression/activity in similar contexts to their phenotypic association.

### Statistical analysis

Univariate analysis was performed using a two-sample *t* test for numerical variables and a Chi-square test for categorical variables. The results have been presented using basic descriptive statistics, such as proportion, median, and range. R version 3.4.1 (ggplots package) was utilised for data analysis and visualisation.

### Ethical approval

The study (ID-51249) was approved by the UK Biobank committee. The UK Biobank has approval from the North West Multi-Centre Research Ethics Committee and the Patient Information Advisory Group. All procedures performed in studies involving human participants were in accordance with the ethical standards of the UK Biobank research committee. Informed consent was obtained from all UK Biobank participants.

### Reporting summary

Further information on research design is available in the Nature Research Reporting Summary linked to this article.

## Supplementary information

Supplementary Information

Reporting Summary

## Data Availability

The UK Biobank data can be retrieved by applying to the UK Biobank (https://www.ukbiobank.ac.uk/register-apply/).

## Code availability

Codes are available from the corresponding author upon reasonable request.
