# Supplementary material for: The role of genetic polymorphisms in endolysosomal ion channels TPC2 and P2RX4 in cancer pathogenesis, prognosis, and diagnosis: a genetic association in the UK Biobank
Source: NPJ Genom Med. 2021 Jul 12;6:58. doi: 10.1038/s41525-021-00221-9 (PMC8275681; doi:10.1038/s41525-021-00221-9)
Supplement: Supplementary file 1 — Supplementary Information [file 41525_2021_221_MOESM1_ESM.pdf]

Supplementary Information

Supplementary Table 1. The associations between endolysosomal ion channel polymorphisms and cancer risk in the UK Biobank population.					
		**Cancer (Cases)**	**Cancer-Free (Controls)**	**OR (95% CI)**	**p-value**
**Total (n)**		83183	385253		
**Female (n)**		47265	206752		
**Male (n)**		35918	178501		
**SNP**	Genotypes (n)				
**rs3750965**	AA	37638	173542		
	GA	36762	170024	1.002(0.99- 1.02)	0.707
	GG	8783	41687	0.97 (0.95-1)	0.027*
**rs35264875**	AA	58604	273995		
	TA	22512	101826	1.03 (1.015-1.05)	0.0001**

	TT	2067	9432	1.02(0.97-1.07)	0 .325
**rs34510004**	AA	0	1	NA	NA
	AG	230	949	1.12 (0.97-1.3)	0.124
	GG	82953	384303		
**rs72932540**	AA	68628	321796		
	GA	13929	60582	1.08 (1.06-1.1)	2.874e-13***
	GG	626	2875	1(0.92-1.1)	0.639
**rs25644**	AA	65014	300861		
	GA	16990	79186	0.99(0.97-1.01)	0.453
	GG	1179	5206	1.05(0.98-1.12)	0.149
**rs28360472**	AA	80430	373147		
	GA	2724	11997	1.05(1-1.1)	0.016*
	GG	29	109	1.23(0.82-1.86)	0.313

Supplementary Table 2. The associations between endolysosomal ion channel polymorphisms and metastasis risk in patients with cancer in the UK Biobank population.					
		Metastatic cancer (Cases)	Primary cancer (Controls)	OR (95% CI)	p-value
Total (n)		334	65559		
Female (n)		174	34187		
Male (n)		160	31372		
SNP	Genotypes(n)				
rs3750965	AA	144	29739		
	GA	145	28931	1.03 (0.78-1.21)	0.82
	GG	45	6889	1.3 (0.97-1.82)	0.097
rs35264875	AA	254	46041		
	TA	73	17864	0.75 (0.58-0.97)	0.028*
	TT	7	1654	0.83 (0.39-1.75)	0.601
rs34510004	AA	0	0	NA	NA

	AG	1	179	1.01(0.15 -7.9)	1
	GG	333	65380		
**rs72932540**	AA	287	54038		
	GA	43	11006	0.73 (0.53-1)	0.071
	GG	4	515	1.53 (0.57-4.12)	0.651
**rs25644**	AA	267	51203		
	GA	61	13435	0.87 (0.66-1.14)	0.365
	GG	6	921	1.28 (0.57-2.89)	0.758
**rs28360472**	AA	319	63409		
	GA	15	2128	1.4 (0.83-2.36)	0.261
	GG	0	22	NA	NA

**Supplementary Table 3. The associations between endolysosomal ion channel polymorphisms and malignancy risk in patients with cancer in the UK Biobank population.**					
		**Malignant Cancer (Cases)**	**Benign Cancer (Controls)**	**OR (95% CI)**	**p-value**
**Total (n)**		66057	969		
**Female (n)**		34511	620		
**Male (n)**		31546	349		
**SNP**	Genotypes(n)				
**rs3750965**	AA	29968	462		
	GA	29136	404	1.1 (0.97-1.26)	0.131
	GG	6953	103	0.99 (0.8-1.22)	0.757
**rs35264875**	AA	46406	682		
	TA	17987	257	1.04 (0.9-1.2)	0.73
	TT	1664	30	0.81 (0.56-1.17)	0.325
**rs34510004**	AA	0	0	NA	NA

	AG	181	1	2.66 (0.37-19)	0.482
	GG	65876	968		
**rs72932540**	AA	54452	804		
	GA	11085	159	1.03 (0.87-1.22)	0.773
	GG	520	6	1.27 (0.57-2.85)	0.677
**rs25644**	AA	51604	736		
	GA	13524	217	0.89 (0.77-1.04)	0.141
	GG	929	16	0.85 (0.52-1.4)	0.547
**rs28360472**	AA	63884	933		
	GA	2151	36	0.87 (0.62-1.22)	0.481
	GG	22	0	NA	NA

Supplementary Table 4. The associations between endolysosomal ion channel polymorphisms and cancer recurrence risk of patients with cancer in the UK Biobank population.					
		**Cancer-occurrence reported once (Controls)**	**Cancer-Recurrence (Cases)**	**OR (95% CI)**	**p-value**
**Total (n)**		62185	20998		
**Female (n)**		34035	13230		
**Male (n)**		28150	7768		
**SNP**	Genotypes(n)				
**rs3750965**	AA	28228	9410		
	GA	27408	9354	1.02(0.99-1.05)	0.166
	GG	6549	2234	1.01(0.96-1.06)	0.406
**rs35264875**	AA	43846	14758		
	TA	16824	5688	1 (0.97-1.04)	0.812
	TT	1515	552	1.08 (0.97-1.19)	0.123

**rs34510004**	AA	0	0	NA	NA
	AG	167	63	1.12(0.84-1.5)	0.5
	GG	62018	20935		
**rs72932540**	AA	51397	17231		
	GA	10336	3593	1.04 (0.99-1.08)	0.091
	GG	452	174	1.14 (0.96-1.36)	0.134
**rs25644**	AA	48566	16448		
	GA	12732	4258	0.98 (0.95-1.03)	0.532
	GG	887	292	0.97 (0.85-1.11)	0.701
**rs28360472**	AA	60146	20284		
	GA	2013	711	1.05 (0.96-1.14)	0.308
	GG	26	3	0.35 (0.1-1.17)	0.103

**Supplementary Table 5. The associations between endolysosomal ion channel polymorphisms and cancer risk for various types of cancer in the UK Biobank population.**						
**1 – Brain cancer**						
			**Brain cancer (Cases)**	**Cancer-free (Controls)**	**OR (95% CI)**	**p-value**
**Total (n)**			502	385253		
**Female (n)**			206	206752		
**Male (n)**			296	178501		
**SNP**	**Gene**	**Genotypes (n)**				
**rs3750965**	TPCN2	AA	245	173542		
		GA	207	170024	0.86(0.72-1.04)	0.128
		GG	50	41687	0.85 (0.62-1.2)	0.329
**rs35264875**	TPCN2	AA	350	273995		
		TA	138	101826	1.06 (0.87-1.28)	0.591
		TT	14	9432	0.86 (0.51-1.47)	0.686

**rs34510004**	TPCN2	AA	0	1	NA	NA
		AG	1	949	0.81 (0.11-5.8)	1
		GG	501	384303		
**rs72932540**	(In close proximity to the *TPCN2* gene)	AA	420	321796		
		GA	80	60582	1.02 (0.8-1.3)	0.973
		GG	2	2875	0.45 (0.11-1.8)	0.521
**rs25644**	P2RX4	AA	395	300861		
		GA	100	79186	0.96 (0.77-1.2)	0.771
		GG	7	5206	1.03 (0.49-2.2)	1
**rs28360472**	P2RX4	AA	480	373147		
		GA	22	11997	1.43 (0.93-2.2)	0.132
		GG	0	109	NA	NA
**2 – Breast cancer**						
			Breast cancer (Cases)	Cancer-free (Controls)	OR (95% CI)	p-value

**Total (n)**			12056	385253		
**Female (n)**			11988	206752		
**Male (n)**			68	178501		
**SNP**	Gene	**Genotypes (n)**				
**rs3750965**	TPCN2	AA	5459	173542		
		GA	5341	170024	1 (0.97-1.04)	0.952
		GG	1256	41687	0.96 (0.9 -1.02)	0.18
**rs35264875**	TPCN2	AA	8440	273995		
		TA	3322	101826	1.06 (1.02-1.1)	0.006**
		TT	294	9432	1 (0.89 -1.12)	0.868
**rs34510004**	TPCN2	AA	0	1	NA	NA
		AG	29	949	0.98 (0.67-1.41)	0.974
		GG	12027	384303		
**rs72932540**		AA	9900	321796		
		GA	2063	60582	1.11(1.05 -1.16)	3.851e-05***

	(In close proximity to the *TPCN2* gene)	GG	93	2875	1.05 (0.85-1.29)	0.675
**rs25644**	P2RX4	AA	9351	300861		
		GA	2539	79186	1.03 (0.99-1.08)	0.174
		GG	166	5206	1.026 (0.88-1.2)	0.778
**rs28360472**	P2RX4	AA	11635	373147		
		GA	418	11997	1.12 (1.01 -1.23)	0.030*
		GG	3	109	0.88 (0.28-2.77)	1
**3 – Bronchus and lung cancer**						
			**Bronchus and lung cancer (Cases)**	**Cancer-free (Controls)**	**OR (95% CI)**	**p-value**
**Total (n)**			2095	385253		
**Female (n)**			949	206752		
**Male (n)**			1146	178501		
**SNP**	**Gene**	**Genotypes (n)**				

**rs3750965**	**TPCN2**	**AA**	941	173542		
		**GA**	934	170024	1.02 (0.93-1.11)	0.797
		**GG**	220	41687	0.97 (0.84 -1.11)	0.746
**rs35264875**	**TPCN2**	**AA**	1467	273995		
		**TA**	582	101826	1.07 (0.97-1.18)	0.192
		**TT**	46	9432	0.89 (0.67-1.2)	0.582
**rs34510004**	**TPCN2**	**AA**	0	1	NA	NA
		**AG**	9	949	1.75 (0.9 -3.4)	0.143
		**GG**	2086	384303		
**rs72932540**	(In close proxi mity to the *TPCN2* gene)	**AA**	1723	321796		
		**GA**	361	60582	1.12 (1-1.3)	0.07
		**GG**	11	2875	0.7 (0.4 -1.3)	0.324
**rs25644**	P2RX4	**AA**	1621	300861		
		**GA**	436	79186	1.02(0.91-1.13)	0.709
		**GG**	38	5206	1.35 (0.98-1.86)	0.079
	P2RX4	**AA**	2014	373147		

**rs28360472**		**GA**	80	11997	1.24(0.99-1.5)	0.074
		**GG**	1	109	1.7 (0.24-12.1)	1
**4 – Colon cancer**						
			Colon cancer (Cases)	Cancer-free (Controls)	OR (95% CI)	p-value
**Total (n)**			2996	385253		
**Female (n)**			1375	206752		
**Male (n)**			1621	178501		
**SNP**	Gene	Genotypes (n)				
**rs3750965**	TPCN2	AA	1348	173542		
		GA	1346	170024	1.03(0.96 -1.11)	0.637
		GG	302	41687	0.92 (0.82 -1.04)	0.289
**rs35264875**	TPCN2	AA	2062	273995		
		TA	859	101826	1.12 (1.03-1.21)	0.005 **
		TT	75	9432	1.023 (0.81 -1.29)	0.685

**rs34510004**	TPCN2	AA	0	1	NA	NA
		AG	5	949	0.68 (0.28-1.63)	0.490
		GG	2991	384303		
**rs72932540**	(In close proxi mity to the *TPCN2* gene)	AA	2489	321796		
		GA	484	60582	1.03 (0.93-1.14)	0.533
		GG	23	2875	1.03 (0.68-1.56)	0.957
**rs25644**	P2RX4	AA	2369	300861		
		GA	583	79186	0.98 (0.9-1.075)	0.154
		GG	44	5206	1.09 (0.8 -1.5)	0.701
**rs28360472**	P2RX4	AA	2922	373147		
		GA	73	11997	0.78(0.62-0.98)	0.038*
		GG	1	109	1.18(0.16-8.45)	1
**5 – Malignant neoplasms, stated or presumed to be primary, of lymphoid, haematopoietic and related tissue**						
			**Malignant neoplasms, stated or presumed to be primary,**	**Cancer-free (Controls)**	**OR (95% CI)**	**p-value**

			**of lymphoid, haematopoietic and related tissue (Cases)**			
**Total (n)**			3666	385253		
**Female (n)**			1592	206752		
**Male (n)**			2074	178501		
**SNP**	Gene	Genotypes (n)				
**rs3750965**	TPCN2	AA	3562	173542		
		GA	102	170024	0.036 (0.03 -0.04)	<2.2e-16 ***
		GG	2	41687	0.0045 (0.001 -0.02)	< 2.2e-16 ***
**rs35264875**	TPCN2	AA	2545	273995		
		TA	1038	101826	1.1(1.02-1.18)	0.013 *
		TT	83	9432	0.92(0.74-1.15)	0.669
**rs34510004**	TPCN2	AA	0	1	NA	NA
		AG	11	949	1.22(0.67-2.21)	0.628

		GG	3655	384303		
**rs72932540**	(In close proxi mity to the *TPCN2* gene)	AA	3037	321796		
		GA	604	60582	1.06 (0.97-1.15)	0.229
		GG	25	2875	0.91 (0.62-1.36)	0.757
**rs25644**	P2RX4	AA	2882	300861		
		GA	741	79186	0.98(0.9-1.06)	0.586
		GG	43	5206	0.87 (0.64-1.17)	0.473
**rs28360472**	P2RX4	AA	3562	373147		
		GA	102	11997	0.89 (0.73-1.08)	0.271
		GG	2	109	1.93(0.48-7.81)	0.659
6- **Malignant neoplasms of lip, oral cavity and pharynx**						
			**Malignant neoplasms of lip, oral cavity and pharynx (Cases)**	**Cancer-free (Controls)**	**OR (95% CI)**	**p-value**
**Total (n)**			918	385253		
**Female (n)**			317	206752		

Male (n)			601	178501		
**SNP**	Gene	Genotypes (n)				
**rs3750965**	TPCN2	AA	413	173542		
		GA	381	170024	0.94(0.82-1.08)	0.42
		GG	124	41687	1.25(1.02-1.52)	0.034*
**rs35264875**	TPCN2	AA	671	273995		
		TA	220	101826	0.88 (0.76-1.03)	0.115
		TT	27	9432	1.18(0.78-1.69)	0.491
**rs34510004**	TPCN2	AA	0	1	NA	NA
		AG	2	949	0.95 (0.15- 2.93)	1
		GG	916	384303		
**rs72932540**	(In close proxi mity to the *TPCN2* gene)	AA	762	321796		
		GA	153	60582	1.07 (0.89-1.27)	0.496
		GG	3	2875	0.46 (0.11- 1.20)	0.207
**rs25644**	P2RX4	AA	698	300861		

		GA	208	79186	1.13(0.97-1.32)	0.126
		GG	12	5206	1.01 (0.54-1.70)	1
**rs28360472**	P2RX4	AA	897	373147		
		GA	21	11997	0.73(0.46-1.10)	0.178
		GG	0	109	NA	NA
**7 – Malignant melanoma**						
			Melanoma cancer (Cases)	Cancer-Free (Controls)	OR (95% CI)	p-value
**Total (n)**			2836	385253		
**Female (n)**			1607	206752		
**Male (n)**			1229	178501		
**SNP**	Gene	Genotypes (n)				
**rs3750965**	TPCN2	AA	1342	173542		
		GA	1204	170024	0.92 (0.85-1)	0.027*

		GG	290	41687	0.9 (0.8 -1.02)	0.103
**rs35264875**	TPCN2	AA	1977	273995		
		TA	787	101826	1.07(0.98-1.16)	0.104
		TT	72	9432	1.04(0.82-1.3)	0.64
**rs34510004**	TPCN2	AA	0	1	NA	NA
		AG	8	949	1.15(0.57-2.3)	0.702
		GG	2828	384303		
**rs72932540**	(In close proxi mity to the *TPCN2* gene)	AA	2309	321796		
		GA	508	60582	1.17 (1.06-1.29)	0.002**
		GG	19	2875	0.92 (0.59-1.4)	0.722
**rs25644**	P2RX4	AA	2254	300861		
		GA	538	79186	0.9(0.82-0.99)	0.042*
		GG	44	5206	1.15 (0.85-1.55)	0.43
**rs28360472**	P2RX4	AA	2748	373147		
		GA	86	11997	0.97(0.78-1.2)	0.806
		GG	2	109	2.5(0.62-10.10)	0.186

8 – Malignant neoplasms of mesothelial and soft tissue						
			**Malignant neoplasms of mesothelial and soft tissue (Cases)**	**Cancer-free (Controls)**	**OR (95% CI)**	**p-value**
**Total (n)**			632	385253		
**Female (n)**			252	206752		
**Male (n)**			380	178501		
**SNP**	Gene	Genotypes (n)				
**rs3750965**	TPCN2	AA	284	173542		
		GA	299	170024	1.14 (0.97-1.33)	0.409
		GG	49	41687	0.69 (0.52-0.93)	0.038*
**rs35264875**	TPCN2	AA	453	273995		
		TA	168	101826	1 (0.85-1.2)	1
		TT	11	9432	0.71(0.39-1.28)	0.308

**rs34510004**	TPCN2	AA	0	1	NA	NA
		AG	0	949	NA	NA
		GG	632	384303		
**rs72932540**	(In close proxi mity to the *TPCN2* gene)	AA	540	321796		
		GA	88	60582	0.87 (0.69-1.09)	0.23
		GG	4	2875	0.85 (0.32-2.27)	0.885
**rs25644**	P2RX4	AA	490	300861		
		GA	133	79186	1.03 (0.85-1.25)	0.791
		GG	9	5206	0.85 (0.44 -1.64)	0.997
**rs28360472**	P2RX4	AA	611	373147		
		GA	19	11997	0.96 (0.61-1.52)	0.978
		GG	2	109	11.22(2.8-45.5)	0.002**
**9 – Malignant neoplasm of the ovary**						
			**Malignant neoplasm of the ovary (Cases)**	**Cancer-free (Controls)**	**OR (95% CI)**	**p-value**

**Total (n)**			1016	385253		
**Female (n)**			1016	206752		
**Male (n)**			0	178501		
**SNP**	Gene	Genotypes (n)				
**rs3750965**	TPCN2	AA	459	173542		
		GA	450	170024	1 (0.89-1.14)	1
		GG	107	41687	0.97 (0.79-1.2)	0.821
**rs35264875**	TPCN2	AA	741	273995		
		TA	255	101826	0.93 (0.8-1.08)	0.307
		TT	20	9432	0.8 (0.51-1.25)	0.330
**rs34510004**	TPCN2	AA	0	1	NA	NA
		AG	3	949	1.2 (0.4-3.7)	1
		GG	1013	384303		
**rs72932540**		AA	852	321796		
		GA	160	60582	1 (0.85-1.2)	1

	(In close proxi mity to the *TPCN2* gene)	GG	4	2875	0.53 (0.2 -1.4)	0.262
**rs25644**	P2RX4	AA	789	300861		
		GA	213	79186	1.03 (0.88-1.2)	0.772
		GG	14	5206	1.02 (0.6-1.73)	1
**rs28360472**	P2RX4	AA	981	373147		
		GA	34	11997	1.08 (0.76-1.5)	0.471
		GG	1	109	3.48 (0.5-25)	0.812
**10 – Malignant neoplasm of prostate**						
			Malignant neoplasm of prostate (Cases)	Cancer-free (Controls)	OR (95% CI)	p-value
**Total (n)**			8025	385253		
**Female (n)**			1	206752		
**Male (n)**			8024	178501		
**SNP**	Gene	Genotypes				

		(n)				
**rs3750965**	TPCN2	AA	3750	173542		
		GA	3494	170024	0.98 (0.93-1.02)	0.036*
		GG	781	41687	0.89 (0.82-0.96)	0.0003***
**rs35264875**	TPCN2	AA	5557	273995		
		TA	2240	101826	1.08 (1.03-1.13)	0.001 **
		TT	228	9432	1.17 (1.02-1.33)	0.011*
**rs34510004**	TPCN2	AA	0	1	NA	NA
		AG	26	949	1.32 (0.89 -1.94)	0.203
		GG	7999	384303		
**rs72932540**	(In close proxi mity to the *TPCN2* gene	AA	6559	321796		
		GA	1390	60582	1.12 (1.06-1.2)	7.76e-05***
		GG	76	2875	1.27 (1.01-1.6)	0.030*
**rs25644**	P2RX4	AA	6246	300861		
		GA	1648	79186	1 (0.95-1.06)	0.941
		GG	131	5206	1.21 (1.02-1.44)	0.035*

**rs28360472**	P2RX4	AA	7763	373147		
		GA	259	11997	1.04 (0.92-1.18)	0.584
		GG	3	109	1.32(0.42 -4.16)	0.885
**11 – Malignant neoplasm of rectum**						
			Malignant neoplasm of rectum (Cases)	Cancer-free (Controls)	OR (95% CI)	p-value
**Total (n)**			1426	385253		
**Female (n)**			500	206752		
**Male (n)**			926	178501		
**SNP**	Gene	Genotypes (n)				
**rs3750965**	TPCN2	AA	641	173542		
		GA	624	170024	0.98 (0.89 -1.1)	0.932
		GG	161	41687	1.05 (0.89 -1.24)	0.645

**rs35264875**	TPCN2	AA	994	273995		
		TA	385	101826	1.03 (0.92-1.16)	0.511
		TT	47	9432	1.36 (1.01-1.82)	0.041*
**rs34510004**	TPCN2	AA	0	1	NA	NA
		AG	3	949	0.85 (0.27 -2.66)	0.995
		GG	1423	384303		
**rs72932540**	(In close proxi mity to the *TPCN2* gene)	AA	1159	321796		
		GA	255	60582	1.13 (0.64 -1.99)	0.027*
		GG	12	2875	1.17 (1.02 -1.34)	0.725
**rs25644**	P2RX4	AA	1112	300861		
		GA	300	79186	1.03 (0.9-1.17)	0.729
		GG	14	5206	0.72 (0.43 -1.23)	0.284
**rs28360472**	P2RX4	AA	1389	373147		
		GA	37	11997	0.83 (0.6 -1.15)	0.292
		GG	0	109	NA	NA

12 – Malignant neoplasm of thyroid gland						
			**Malignant neoplasm of thyroid gland (Cases)**	**Cancer-free (Controls)**	**OR (95% CI)**	**p-value**
**Total (n)**			495	385253		
**Female (n)**			375	206752		
**Male (n)**			120	178501		
**SNP**	Gene	Genotypes (n)				
**rs3750965**	TPCN2	AA	232	173542		
		GA	210	170024	0.93 (0.78-1.11)	0.434
		GG	53	41687	0.99 (0.74-1.31)	0.912
**rs35264875**	TPCN2	AA	361	273995		
		TA	116	101826	0.85 (0.69-1.05)	0.19
		TT	18	9432	1.5 (0.94-2.41)	0.162
**rs34510004**	TPCN2	AA	0	1	NA	NA
		AG	1	949	0.8 (0.11-5.71)	1

		GG	494	384303		
**rs72932540**	(In close proximity to the *TPCN2* gene)	AA	407	321796		
		GA	81	60582	1.05 (0.83 -1.33)	0.693
		GG	7	2875	1.91 (0.9-4.03)	0.138
**rs25644**	P2RX4	AA	392	300861		
		GA	96	79186	0.93 (0.74-1.16)	0.564
		GG	7	5206	1.05 (0.5-2.21)	1
**rs28360472**	P2RX4	AA	474	373147		
		GA	21	11997	1.4 (0.89 -2.13)	0.189
		GG	0	109	NA	NA
**13 – Malignant neoplasm of urinary tract**						
			Cancer cases	Cancer-free Controls	OR (95% CI)	p-value
**Total (n)**			2124	385253		
**Female (n)**			618	206752		
**Male (n)**			1506	178501		

**SNP**	Gene	Genotypes (n)				
**rs3750965**	TPCN2	AA	942	173542		
		GA	947	170024	1.02 (0.93-1.11)	0.592
		GG	235	41687	1.025(0.89-1.17)	0.631
**rs35264875**	TPCN2	AA	1520	273995		
		TA	545	101826	1.14(0.88-1.48)	0.49
		TT	59	9432	0.96(0.87 -1.06)	0.405
**rs34510004**	TPCN2	AA	0	1	NA	NA
		AG	6	949	1.15 (0.51 -2.56)	0.908
		GG	2118	384303		
**rs72932540**	(In close proxi mity to the *TPCN2* gene)	AA	1786	321796		
		GA	316	60582	0.94 (0.83 -1.06)	0.325
		GG	22	2875	1.4 (0.91-2.1)	0.17
**rs25644**	P2RX4	AA	1627	300861		
		GA	466	79186	1.09 (0.98 -1.2)	0.115

		GG	31	5206	1.08 (0.76 -1.54)	0.664
**rs28360472**	P2RX4	AA	2054	373147		
		GA	69	11997	1.04 (0.82-1.3)	0.768
		GG	1	109	1.7 (0.23 – 11.93)	1

Supplementary Table 6. The associations between endolysosomal ion channel polymorphisms and cancer recurrence risk in the UK Biobank population.

1 – Brain cancer

			**Cancer recurrence in patients with brain cancer (Cases)**	**Cancer occurrence in patients with Brain Cancer (Controls)**	**OR (95 %CI)**	**p-value**
**Total (n)**			72	430		
**Female (n)**			35	171		
**Male (n)**			37	259		
**SNP**	Gene	Genotypes				

		(n)				
**rs3750965**	TPCN2	AA	30	215		
		GA	31	176	1.26 (0.74-2.16)	0.479
		GG	11	39	2.02(0.94-4.4)	0.111
**rs35264875**	TPCN2	AA	53	297		
		TA	18	120	0.86 (0.49-1.53)	0.653
		TT	1	13	0.45(0.058-3.51)	0.658
**rs34510004**	TPCN2	AA	0	0	NA	NA
		AG	0	1	NA	NA
		GG	72	429		
**rs72932540**	(In close proximity to the *TPCN2* gene)	AA	60	360		
		GA	11	69	0.94 (0.47-1.9)	1
		GG	1	1	5.64(0.35-91.22)	0.671
**rs25644**	*P2RX4*	AA	55	340		
		GA	16	84	1.18 (0.64-2.15)	0.712

		GG	1	6	1 (0.12-8.4)	1
**rs28360472**	*P2RX4*	AA	67	413		
		GA	5	17	1.8 (0.65-5.1)	0.403
		GG	0	0	NA	NA
**2 – Breast Cancer**						
			**Cancer recurrence in patients with breast cancer (Cases)**	**Cancer occurrence in patients with breast cancer (Controls)**	**OR (95 %CI)**	**p-value**
**Total (n)**			2873	9183		
**Female (n)**			2856	9132		
**Male (n)**			17	51		
**SNP**	Gene	Genotypes (n)				
**rs3750965**	TPCN2	AA	1282	4177		
		GA	1309	4032	1.06 (0.97-1.16)	0.221

		GG	282	974	0.94 (0.81-1.09)	0.457
**rs35264875**	TPCN2	AA	2003	6437		
		TA	798	2524	1.014 (0.92- 1.11)	0.758
		TT	72	222	1.04 (0.79-1.36)	0.818
**rs34510004**	TPCN2	AA	0	0		
		AG	4	25	0.51 (0.18-1.47)	0.293
		GG	2869	9158		
**rs72932540**	(In close proximity to the *TPCN2* gene)	AA	2356	7544		
		GA	492	1571	1 (0.9-1.12)	0.983
		GG	25	68	1.18 (0.74-1.86)	0.567
**rs25644**	*P2RX4*	AA	2253	7098		
		GA	579	1960	0.93 (0.84-1.03)	0.185
		GG	41	125	1.05 (0.74-1.5)	0.929
**rs28360472**	*P2RX4*	AA	2773	8862		
		GA	99	319	0.99 (0.79-1.25)	0.991

		GG	1	2	1.6 (0.14-17.63)	1
**3 – Bronchus and lung cancer**						
			**Cancer recurrence in patients with bronchus and lung cancer (Cases)**	**Cancer occurrence in patients with bronchus and l ung cancer (Controls)**	**OR (95 %CI)**	**p-value**
**Total (n)**			327	1768		
**Female (n)**			154	795		
**Male (n)**			173	973		
**SNP**	Gene	Genotypes (n)				
**rs3750965**	TPCN2	AA	144	797		
		GA	143	791	1 (0.78-1.29)	1
		GG	40	180	1.24 (0.84-1.8)	0.342
**rs35264875**	TPCN2	AA	241	1226		

		TA	82	500	0.84 (0.64-1.09)	0.214
		TT	4	42	0.48 (0.17-1.4)	0.231
**rs34510004**	TPCN2	AA	0	0		
		AG	1	8	0.67 (0.08-5.41)	1
		GG	326	1760		
**rs72932540**	(In close proximity to the *TPCN2* gene)	AA	269	1454		
		GA	56	305	0.99 (0.72-1.4)	1
		GG	2	9	1.2 (0.26-5.6)	1
**rs25644**	*P2RX4*	AA	242	1379		
		GA	77	359	1.2 (0.92-1.6)	0.185
		GG	8	30	1.5 (0.67-3.4)	0.416
**rs28360472**	*P2RX4*	AA	311	1703		
		GA	16	64	1.37 (0.78-2.4)	0.345
		GG	0	1	NA	NA
**4 – Colon cancer**						

			Cancer recurrence in patients with colon cancer (Cases)	Cancer-occurrence in patients with colon cancer (Controls)	OR (95 %CI)	p-value
**Total (n)**			582	2414		
**Female (n)**			255	1120		
**Male (n)**			327	1294		
**SNP**	Gene	Genotypes (n)				
**rs3750965**	TPCN2	AA	246	1102		
		GA	275	1071	1.15 (0.95-1.39)	0.166
		GG	61	241	1.13 (0.83-1.55)	0.481
**rs35264875**	TPCN2	AA	389	1673		
		TA	176	683	1.1 (0.9-1.34)	0.337
		TT	17	58	1.26 (0.73-2.2)	0.5
**rs34510004**	TPCN2	AA	0	0		

		AG	0	5		
		GG	582	2409		
**rs72932540**	(In close proximity to the *TPCN2* gene)	AA	467	2022		
		GA	108	376	1.24 (0.98-1.58)	0.081
		GG	7	16	1.89 (0.77-4.63)	0.248
**rs25644**	*P2RX4*	AA	472	1897		
		GA	102	481	0.85 (0.67-1.08)	0.205
		GG	8	36	0.89 (0.41-1.9)	0.923
**rs28360472**	*P2RX4*	AA	572	2350		
		GA	10	63	0.65 (0.33-1.28)	0.27
		GG	0	1	NA	NA
**5 – Malignant neoplasms, stated or presumed to be primary, of lymphoid, haematopoietic and related tissue**						
			**Cancer recurrence in patients with**	**Cancer occurrence in patients with malignant neoplasms,**	**OR (95 %CI)**	**p-value**

			malignant neoplasms, stated or presumed to be primary, of lymphoid, haematopoietic and related tissue (Cases)	stated or presumed to be primary, of lymphoid, haematopoietic and related tissue (Controls)		
**Total (n)**			917	2749		
**Female (n)**			369	1223		
**Male (n)**			548	1526		
**SNP**	Gene	Genotypes (n)				
**rs3750965**	TPCN2	AA	434	1252		
		GA	393	1212	0.94 (0.8 -1.1)	0.43
		GG	90	285	0.91 (0.7 -1.2)	0.525
**rs35264875**	TPCN2	AA	627	1918		

		TA	267	771	1.06 (0.9-1.25)	0.523
		TT	23	60	1.17(0.72-1.9)	0.610
**rs34510004**	TPCN2	AA	0	0		
		AG	2	9	0.67 (0.14-3.1)	0.861
		GG	915	2740		
**rs72932540**	(In close proximity to the *TPCN2* gene)	AA	749	2288		
		GA	161	443	1.11 (0.91-1.35)	0.326
		GG	7	18	1.19 (0.49-2.8)	0.879
**rs25644**	P2RX4	AA	703	2179		
		GA	199	542	1.14 (0.94-1.37)	0.182
		GG	15	28	1.66 (0.88-3.13)	0.159
**rs28360472**	P2RX4	AA	892	2670		
		GA	25	77	0.97 (0.62-1.54)	0.995
		GG	0	2	NA	NA

6- Malignant neoplasms of lip, oral cavity and pharynx						
			**Cancer recurrence in patients with malignant neoplasms of lip, oral cavity and pharynx (Cases)**	**Cancer occurrence in patients with malignant neoplasms of lip, oral cavity and pharynx (Controls)**	**OR (95 %CI)**	**p-value**
**Total (n)**			229	689		
**Female (n)**			78	239		
**Male (n)**			151	450		
**SNP**	Gene	Genotypes (n)				
**rs3750965**	TPCN2	AA	98	315		
		GA	106	275	1.24 (0.90-1.71)	0.216
		GG	25	99	0.81 (0.49-1.32)	0.4794
**rs35264875**	TPCN2	AA	163	508		

		TA	57	163	1.09 (0.76-1.54)	0.695
		TT	9	18	1.57 (0.66-3.50)	0.4
**rs34510004**	TPCN2	AA	0	0	NA	NA
		AG	0	2	NA	NA
		GG	229	687		
**rs72932540**	(In clos e proxi mity to the *TPCN2* gene)	AA	189	573		
		GA	39	114	1.04 (0.69- 1.54)	0.939
		GG	1	2	1.61 (0.05-20.02)	1
**rs25644**	*P2RX4*	AA	168	530		
		GA	60	148	1.28 (0.90-1.80)	0.193
		GG	1	11	0.32 (0.013-1.70)	0.354
**rs28360472**	*P2RX4*	AA	221	676		
		GA	8	13	1.89 (0.73-4.60)	0.249
		GG	0	0	NA	NA
**7 – Malignant melanoma**						

			Cancer recurrence in malignant melanoma (Cases)	Cancer occurrence in patients with malignant melanoma (Controls)	OR (95 %CI)	p-value
**Total (n)**			778	2058		
**Female (n)**			429	1178		
**Male (n)**			349	880		
**SNP**	Gene	Genotypes (n)				
**rs3750965**	TPCN2	AA	380	962		
		GA	325	879	0.93 (0.79-1.1)	0.484
		GG	73	217	0.85 (0.64-1.1)	0.312
**rs35264875**	TPCN2	AA	524	1453		
		TA	232	555	1.2 (0.97-1.4)	0.125
		TT	22	50	1.2 (0.7-2)	0.53
**rs34510004**	TPCN2	AA	0	0	NA	NA

		AG	2	6	0.88 (0.18- 4.4)	1
		GG	776	2052		
**rs72932540**	(In close proximity to the *TPCN2* gene)	AA	620	1689		
		GA	152	356	1.16 (0.94-1.43)	0.177
		GG	6	13	1.22 (0.46-3.22)	0.839
**rs25644**	P2RX4	AA	618	1636		
		GA	149	389	1.02 (0.82-1.25)	0.94
		GG	11	33	0.88 (0.44-1.8)	0.853
**rs28360472**	*P2RX4*	AA	756	1992		
		GA	21	65	0.85 (0.52-1.4)	0.61
		GG	1	1	2.65 (0.17-42.4)	1
**8 – Malignant neoplasms of mesothelial and soft tissue**						
			**Cancer recurrence in patients with**	**Cancer occurrence in patients with malignant neoplasms of**	**OR (95 %CI)**	**p-value**

			malignant neoplasms of mesothelial and soft tissue (Cases)	mesothelial and soft tissue (Controls)		
**Total (n)**			114	518		
**Female (n)**			46	206		
**Male (n)**			68	312		
**SNP**	Gene	Genotypes (n)				
**rs3750965**	TPCN2	AA	43	241		
		GA	60	239	1.4 (0.9-2)	0.147
		GG	11	38	1.62 (0.78-3.4)	0.284
**rs35264875**	TPCN2	AA	84	369		
		TA	28	140	0.88 (0.55-1.4)	0.673
		TT	2	9	0.97 (0.21-4.6)	1
**rs34510004**	TPCN2	AA	0	0		

		AG	0	0		
		GG	114	518		
**rs72932540**	(in close proximity to the *TPCN2* gene)	AA	101	439		
		GA	11	77	0.62 (0.31-1.2)	0.208
		GG	2	2	4.4 (0.62-31.23)	0.342
**rs25644**	*P2RX4*	AA	89	401		
		GA	25	108	1.04 (0.64-1.7)	0.967
		GG	0	9	NA	NA
**rs28360472**	*P2RX4*	AA	112	499		
		GA	2	17	0.53 (0.12-2.3)	0.570
		GG	0	2	NA	NA
**9 – Malignant neoplasm of the ovary**						
			**Cancer recurrence in patients with malignant**	**Cancer occurrence in patients with**	**OR (95 %CI)**	**p-value**

			neoplasm of the ovary (Cases)	malignant neoplasm of the ovary (Controls)		
**Total (n)**			224	792		
**Female (n)**			224	792		
**Male (n)**			0	0		
**SNP**	Gene	Genotypes (n)				
**rs3750965**	TPCN2	AA	97	362		
		GA	106	344	1.15 (0.84-1.57)	0.425
		GG	21	86	0.911 (0.54-1.5)	0.831
**rs35264875**	TPCN2	AA	158	583		
		TA	58	197	1.08 (0.77-1.5)	0.699
		TT	8	12	2.4 (0.97-6)	0.085
**rs34510004**	TPCN2	AA	0	0		
		AG	2	1	7.16 (0.64-79)	0.242

		GG	222	791		
**rs72932540**	(In close proximity to the *TPCN2* gene)	AA	191	661		
		GA	32	128	0.86 (0.57-1.3)	0.567
		GG	1	3	1.15 (0.12 -11.2)	1
**rs25644**	*P2RX4*	AA	183	606		
		GA	36	177	0.67 (0.45-0.99)	0.060
		GG	5	9	1.84 (0.6 -5.6)	0.436
**rs28360472**	*P2RX4*	AA	215	766		
		GA	9	25	1.28 (0.59-2.8)	0.675
		GG	0	1		
**10 – Prostate cancer**						
			**Cancer recurrence in patients with prostate cancer (Cases)**	**Cancer occurrence in patients with prostate cancer (Controls)**	**OR (95 %CI)**	**p-value**

**Total (n)**			1189	6836		
**Female (n)**			0	1		
**Male (n)**			1189	6835		
**SNP**	Gene	Genotypes (n)				
**rs3750965**	TPCN2	AA	529	3221		
		GA	525	2969	1.08 (0.94-1.2)	0.282
		GG	135	646	1.27 (1 -1.5)	0.026*
**rs35264875**	TPCN2	AA	823	4734		
		TA	328	1912	0.98 (0.85-1.13)	0.878
		TT	38	190	1.15 (0.81-1.65)	0.498
**rs34510004**	TPCN2	AA	0	0	NA	NA
		AG	6	20	1.7 (0.7-4.3)	0.362
		GG	1183	6816		
**rs72932540**	(In clos e proxi mity to	AA	969	5590		
		GA	203	1187	0.98 (0.83-1.15)	0.904

	the *TPCN2* gene)	GG	17	59	1.67 (0.97-2.9)	0.091
**rs25644**	*P2RX4*	AA	938	5308		
		GA	240	1408	0.97 (0.84-1.14)	0.673
		GG	11	120	0.522 (0.28-0.97)	0.047
**rs28360472**	P2RX4	AA	1156	6607		
		GA	33	226	0.83 (0.58-1.21)	0.385
		GG	0	3	NA	NA
**11– Malignant neoplasm of rectum**						
			**Cancer recurrence in patients with malignant neoplasm of rectum (Cases)**	**Cancer occurrence in patients with rectum cancer (Controls)**	**OR (95 %CI)**	**p-value**
**Total (n)**			281	1145		
**Female (n)**			101	399		

Male (n)			180	746		
**SNP**	**Gene**	Genotypes (n)				
**rs3750965**	TPCN2	AA	113	528		
		GA	141	483	1.4 (1.06-1.8)	0.033*
		GG	27	134	0.94 (0.59 -1.5)	0.888
**rs35264875**	TPCN2	AA	195	799		
		TA	80	305	1.1 (0.82-1.5)	0.682
		TT	6	41	0.59 (0.25-1.4)	0.330
**rs34510004**	TPCN2	AA	0	0	NA	NA
		AG	1	2	2.04 (0.18-22.6)	1
		GG	280	1143		
**rs72932540**	(In close proximity to the *TPCN2* gene)	AA	228	931		
		GA	52	203	1.05 (0.75-1.48)	0.862
		GG	1	11	0.37 (0.05-2.9)	0.536

**rs25644**	P2RX4	AA	227	885		
		GA	52	248	0.82 (0.59-1.15)	0.268
		GG	2	12	0.65 (0.14-3)	0.817
**rs28360472**	P2RX4	AA	273	1116		
		GA	8	29	1.13 (0.51-2.5)	0.930
		GG	0	0	NA	NA
**12 – Malignant neoplasm of thyroid gland**						
			**Cancer recurrence in patients with malignant neoplasm of thyroid gland (Cases)**	**Cancer occurrence in patients with malignant neoplasm of thyroid gland (Controls)**	OR (95 %CI)	p-value
**Total (n)**			135	360		
**Female (n)**			109	266		
**Male (n)**			26	94		
**SNP**	Gene	Genotypes				

		(n)				
**rs3750965**	TPCN2	AA	72	160		
		GA	54	156	0.77 (0.5 -1.2)	0.258
		GG	9	44	0.45 (0.2 -1)	0.060
**rs35264875**	TPCN2	AA	96	265		
		TA	34	82	1.14 (0.72-1.81)	0.651
		TT	5	13	1.06 (0.36-3)	1
**rs34510004**	TPCN2	AA	0	0	NA	NA
		AG	1	0	NA	NA
		GG	134	360		
**rs72932540**	(In close proximity to the *TPCN2* gene)	AA	106	301		
		GA	26	55	1.34 (0.8-2.22)	0.326
		GG	3	4	2.1 (0.47-9.7)	0.57
**rs25644**	P2RX4	AA	104	288		
		GA	29	67	1.2 (0.73 -1.95)	0.550

		GG	2	5	1.07 (0.2-5.57)	1
**rs28360472**	P2RX4	AA	129	345		
		GA	6	15	1.07 (0.41-2.82)	1
		GG	0	0	NA	NA
**13 – Malignant neoplasm of urinary tract**						
			**Cancer recurrence in malignant neoplasms of urinary tract (Cases)**	**Cancer occurrence in patients with malignant neoplasms of urinary tract (Controls)**	OR (95 %CI)	p-value
**Total (n)**			532	1592		
**Female (n)**			145	473		
**Male (n)**			387	1119		
**SNP**	Gene	Genotypes (n)				
**rs3750965**	TPCN2	AA	255	687		

		GA	226	721	0.84 (0.69-1.04)	0.122
		GG	51	184	0.75 (0.53-1.1)	0.111
**rs35264875**	TPCN2	AA	367	1153		
		TA	149	396	1.18 (0.94-1.47)	0.156
		TT	16	43	1. 2 (0.62-2)	0.713
**rs34510004**	TPCN2	AA	0	0	NA	NA
		AG	2	4	1.5 (0.27-8.2)	1
		GG	530	1588		
**rs72932540**	(In close proximity to the *TPCN2* gene)	AA	438	1348		
		GA	85	231	1.12 (0.85-1.47)	0.407
		GG	9	13	2.1 (0.9-5.01)	0.128
**rs25644**	P2RX4	AA	405	1222		
		GA	117	349	1 (0.79-1.27)	0.973
		GG	10	21	1.43 (0.67-3.06)	0.466
**rs28360472**	P2RX4	AA	518	1536		

		GA	14	55	0.76 (0.42 -1.37)	0.431
		GG	0	1	NA	NA

**Supplementary Table 7. Allele Frequencies and Hardy Weinberg equilibrium of endolysosomal ion channel polymorphisms**							
**SNP**	Gene	Type	Location (GRCh38)	Alleles	Global MAF	MAF in our population	HWE (P-value)
**rs3750965**	TPCN2	SNV	11:69072692	A>C,G	G=0.31	G=0.33	0.65
**rs35264875**	TPCN2	SNV	11:69078931	A>C,T	T=0.17	T=0.16	0.34
**rs34510004**	TPCN2	SNV	11:69081448	G>A	A=0.00102	A=0.0013	0.77
**rs3829241**	TPCN2	SNV	11:69087895	G>A	A=0.359398	A=0.39	0
**rs72932540**	In close proximity to the *TPCN2* gene	SNV	11:69154575	A>G	G=0.0951	G=0.09	0.41
**rs25644**	P2RX4	SNV	12:121228843	A>G	G=0.121560	G=0.12	0.47
**rs28360472**	P2RX4	SNV	12:121232473	A>G	G=0.01337	G=0.02	0.095
Ncbi.nlm.nih.gov. 2020. *Home - SNP - NCBI*							
